# Electronic Structure of Low-Temperature Solution-Processed Amorphous Metal Oxide Semiconductors for Thin-Film Transistor Applications

**DOI:** 10.1002/adfm.201404375

**Published:** 2015-02-18

**Authors:** Josephine Socratous, Kulbinder K Banger, Yana Vaynzof, Aditya Sadhanala, Adam D Brown, Alessandro Sepe, Ullrich Steiner, Henning Sirringhaus

**Affiliations:** Cavendish Laboratory19 JJ Thomson Avenue, CB3 OHE, Cambridge, UK

**Keywords:** indium–zinc oxide, nitrates, solution-processing, spectroscopy, subgap density of states

## Abstract

The electronic structure of low temperature, solution-processed indium–zinc oxide thin-film transistors is complex and remains insufficiently understood. As commonly observed, high device performance with mobility >1 cm^2^ V^−1^ s^−1^ is achievable after annealing in air above typically 250 °C but performance decreases rapidly when annealing temperatures ≤200 °C are used. Here, the electronic structure of low temperature, solution-processed oxide thin films as a function of annealing temperature and environment using a combination of X-ray photoelectron spectroscopy, ultraviolet photoelectron spectroscopy, and photothermal deflection spectroscopy is investigated. The drop-off in performance at temperatures ≤200 °C to incomplete conversion of metal hydroxide species into the fully coordinated oxide is attributed. The effect of an additional vacuum annealing step, which is beneficial if performed for short times at low temperatures, but leads to catastrophic device failure if performed at too high temperatures or for too long is also investigated. Evidence is found that during vacuum annealing, the workfunction increases and a large concentration of sub-bandgap defect states (re)appears. These results demonstrate that good devices can only be achieved in low temperature, solution-processed oxides if a significant concentration of acceptor states below the conduction band minimum is compensated or passivated by shallow hydrogen and oxygen vacancy-induced donor levels.

## 1. Introduction

Over the last decade, there has been an increasing scientific interest in amorphous metal oxide semiconductors (MOS) for thin-film transistor applications. Oxide TFTs provide higher performance than amorphous silicon devices with mobilities μ>10cm^2^ V^−1^ s^−1^ and better threshold voltage stability making them attractive candidates, in particular, for driving active matrix organic light-emitting diode displays.^1^,^2^ Furthermore, these materials are transparent and their applications can be extended to circuits on windows of vehicles or buildings.^3^ Most of the research so far, has focused on oxides deposited via low-temperature sputtering techniques and a wide range of ternary and quaternary elemental compositions has been explored with InGaZnO (IGZO) being one of the best performing materials.^4^–^8^ Bonding in these materials is ionic; the metal atoms are positively charged by donating electrons to the oxygen. The closed 2*p* shell of the oxygen anions forms the valence band maximum (VBM) and the conduction band minimum (CBM) mainly comprises the empty spherical orbitals of the metal cations. In comparison with the isolated atoms, the energy of the oxygen 2*p*- and the metal *s*-orbitals is shifted as a result of the electrostatic, so-called Madelung potential associated with the positive and negative ions, lowering the energy of the filled oxygen 2*p*-orbitals and raising the energy of the empty metal *s*-orbitals, which results in a high bandgap.^9^,^10^

Significant research focus has been on better understanding the complex chemistry and electronic structure of defects in low-temperature processed amorphous MOS. A well-researched, common defect in these materials is oxygen vacancies. These involve the nonbonding state of four metal cations (each contributing 1/2 electron) caused by a missing nearby oxygen atom. The dangling bonds of these four electrons combine to form a symmetric a_1_ state, which lies in the bandgap and three states above the CB. The vacancy *V*_o_ can be neutral *V*_o_^0^, singly ionized *V*_o_^+1^ or doubly ionized *V*_o_^2+^. The different lattice relaxations associated with the three charge states changes their energetic position in the bandgap and in their equilibrium configurations, the neutral one lies close to VBM and *V*_o_^2+^ closer to CBM.^11^ When the Fermi level *E_f_* is close to the conduction band, *V*_o_^0^ has the lowest formation energy and it is thus, the most likely to form in n-type oxides. This is further supported by density functional theory calculations, which have revealed that in equilibrium conditions, oxygen vacancies form fully-occupied states mostly near the VBM and less often near the CBM.^9^–^14^ Oxygen vacancies have been observed experimentally by combining photoluminescence and electron spin resonance and by hard X-ray photoemission spectroscopy.^14^–^18^ It was suggested that oxygen vacancies generated by removing individual oxygen atoms from the lattice mainly produce deep, localized, and filled states near the VBM. States close to the VBM are fully occupied and therefore do not influence electron transport. However, upon thermal annealing structural equilibration, results in more delocalized oxygen vacancy configurations that then act as shallow donors.^12^ Furthermore, it has been reported that the missing oxygen atom could be substituted by a hydrogen atom.^19^ The substitutional hydrogen *H*_o_, bonds equally to the four metal cations and forms a bonding state deep in the VB, which is occupied by the two electrons from the four metal cations. The third electron, coming from the hydrogen atom, occupies one of the antibonding states that lie above the conduction band. This electron is then transferred to the CBM making this complex acting as a shallow donor.

More generally, H^+^ has the lowest formation energy throughout the whole bandgap of ZnO and similar materials; thus, hydrogen always acts as a shallow donor donating electrons to the CB.^20^,^21^ Interstitial hydrogen, *H*_i_ forms an O–H bond either along the direction of the original O-metal bond (bond-center BC configuration) or opposite to it (antibonding AB configuration). Similarly, to substitutional hydrogen, *H*_o_ discussed above, *H*_i_ also acts as a donor with free electrons expected to be generated according to H + –O^2−^– (in network) → –OH^−^ + e^−^.^20^,^22^,^23^ It was concluded that the donated electron donated to the CB is free and hence, that interstitial hydrogen produces donor levels, which in their stable state, are coordinated by three metal cations and have a formation energy of ≈0.45 eV.^12^

Electrons from such hydrogen or oxygen vacancy-induced donors might be responsible for filling a density of acceptor states below the CBM, which could act as electron traps if they remain unfilled. Such states were recently reported by device simulations at ≈0.8 eV below the CBM with a relatively low density of states (≈10^16^–10^17^ cm^−3^ eV^−1^).^9^,^10^ In the presence of a sufficiently high concentration of shallow donors, these acceptor states may be compensated by electrons from the donors and electron transport may not be significantly affected. In fact, it was also reported that hydrogen could directly passivate defects and prevent them to act as electron traps. An example of such a complex is a zinc vacancy *V*_Zn_ substituted by two hydrogen atoms (H–II).^22^,^24^ Zinc vacancies are known to form acceptor-like states close to the VBM. In n-type oxides, these states have the lowest formation energy among the native point defects and are expected to occur in modest concentrations.^13^ These acceptor states are believed to be associated with a green luminescence centered around 2.2 eV. Doping with hydrogen was shown to suppress this luminescence, indicating that hydrogen acts as a passivating agent for *V*_Zn_.^22^,^24^ Shallow hydrogen donors have been observed experimentally by resolving the hyperfine structure of the H nucleus in electron nuclear double resonance experiments.^25^ The complex behavior of hydrogen atoms which create molecular units with different coordination numbers and structures, makes it difficult to experimentally distinguish between the different defects. It was in fact reported that the complexes formed are strongly dependent on the source of the starting material, which further adds to the complexity.^26^ Metal impurities, such as trivalent Al_3_^+^, may also act as dopants either on their own or by bonding with hydrogen. 22,27

As mentioned above, the main technique for oxide deposition is sputtering. Although this is a generally low cost and powerful low temperature deposition technique, there may be certain limitations, for example, in ensuring large-area compositional uniformity. Preferential sputtering of one of the elements in an originally fixed composition sputter target can alter the composition of the target, which becomes a challenge when depositing films for large-area displays. As a result, there has been a growing interest in low temperature solution-processing approaches for amorphous MOS. This is highly dependent on suitable metal-organic precursors for oxide formation. The chemistries of the first precursors^28^–^31^ required high annealing temperatures (>300 °C) for the oxide framework to fully develop. In 2011, our group in collaboration with Panasonic reported an approach based on metal alkoxide precursors, which allowed solution-processed TFTs with performance comparable with sputtered TFTs at low process temperatures of 230–250 °C, close to being compatible with the plastic substrates used in flexible electronics.^32^ Alternative low temperature routes include the use of exothermic combustion processing on the substrate.^33^ Most recently, a route based on commercially available nitrates has produced TFTs with good performance at low temperatures.^34^ In this route, the metal nitrate precursors are dissolved in water and the metal cations form metal hydroxide species in solution, which are then converted into the fully coordinated oxide during annealing of the films. This hydrolysis reaction releases many hydrogen ions H^+^ that could stay incorporated in the film in the ways described above. The nitrate ions are not bonded to the hydroxide complexes but are rather dispersed and as recently reported, they can induce mobile H^+^ ions.^34^,^35^ The films produced from nitrate precursors are therefore potentially rich in H^+^. A common feature of the different solution-processing routes is that device performance drops dramatically to unusable levels if annealing temperatures much below 200–250 °C are used; the reasons for this degradation are generally not well understood and the corresponding defect states have not been identified.

In this work, we use the recently reported nitrate precursor route to better understand the evolution of the thin film electronic structure as the deposited metal hydroxide film is converted into the fully coordinated oxide during thermal annealing. We are particularly interested in understanding the effect of vacuum annealing that has recently been claimed to enable a significant reduction of the process temperature.^34^ We focus in this work on simple thermal annealing and do not investigate combined thermal and ultraviolet light exposure treatments that may enable further reduced process temperatures.^36^ We use X-ray photoelectron spectroscopy (XPS), ultraviolet photoelectron spectroscopy (UPS), and photothermal deflection spectroscopy (PDS) to gain information on the electronic density of states in the bandgap for different processing conditions. In particular, PDS is a very powerful technique for measuring very sensitively the optical absorption due to defect states within the bandgap of the semiconducting oxides. Our results provide new insight into the complex electronic structure of these low temperature, solution-processed materials, and point in particular to the crucial importance of hydrogen-induced shallow donor states that are needed to compensate acceptor states below the CBM and to achieve high device performance.

## 2.  Results and Discussion

### 2.1.  Effect of Annealing Temperature in Ambient Air

**Figure 1:**
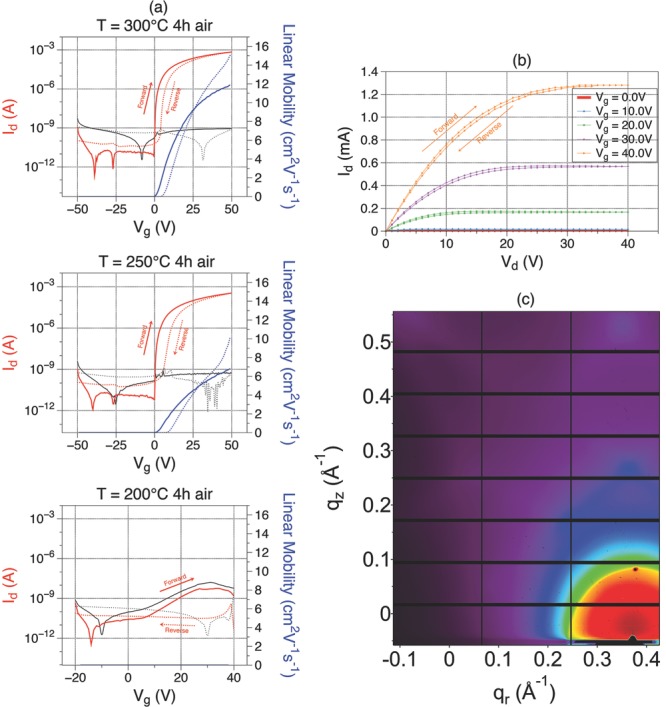
a) Red: drain current. Black: gate leakage current. Blue: Linear Mobility. Solid lines represent forward scan. Dotted lines represent reverse scan. Transfer curves for IZO (In_2_O_3_:ZnO = 6:4) TFTs annealed in air for 4 h at different temperatures (Channel length/width 300/3000 μm; drain voltage *V*_d_ = 5 V). b) Output curve for a device annealed in air for 4 h at 300 °C. c) GIWAXS image for a ternary oxide film with composition In_2_O_3_:ZnO = 6:4, which was annealed in air at 400 °C for 4 h.

**Figure** **1** shows TFT characteristics for an IZO semiconducting layer in the ratio of In_2_O_3_:ZnO = 6:4. Details of the fabrication techniques can be found in the Experimental Section and in Figure S1 (Supporting Information). A grazing incidence wide angle X-ray scattering (GIWAXS) image of a film annealed in air for 4 h at 400 °C is shown in Figure 1c, which confirms the amorphous nature of this film (no peaks are observed) even at this high annealing temperature. In fact, all films discussed in this paper, including the ones processed at lower temperatures, were found to be amorphous. Figure 1a shows transfer curves for samples prepared at the same time and annealed in air for 4 h at different temperatures. A mobility of 12 cm^2^ V^−1^ s^−1^ was obtained for devices annealed at 300 °C. This dropped to 7 cm^2^ V^−1^ s^−1^ at 250 °C. The On–off ratio was larger than 10^7^, the onset voltage –0.5 < *V*_on_< 0.5 V, and the subthreshold slope <1 V dec^−1^. Figure S2 (Supporting Information) shows the spread in mobility when different devices prepared under the same conditions are compared. A mean mobility of 11.3 ± 6.5 cm^2^ V^−1^ s^−1^ and 5.5 ± 1.7 cm^2^ V^−1^ s^−1^ is obtained at 300 and 275 °C, respectively. Figure S3 (Supporting Information) shows that TFT characteristics could still be observed at 215 °C, although the mobility is very low (<0.1 cm^2^ V^−1^ s^−1^). At 200 °C (Figure 1), the drain current falls below the gate leakage and the TFTs stop working completely, even when the films are annealed overnight for 12 h (Figure S4, Supporting Information). The sharp drop in drain current and poor TFT performance at this temperature is in agreement with the recent report on nitrate precursors, where this deterioration is attributed to incomplete decomposition of the precursor.^34^ Also, the hysteresis in the device characteristics was found to increase with decreasing annealing temperature. Hysteresis generally depends on experimental conditions and possibly on the surface contamination, since the films are only ≈10-nm thick. It can also occur due to electron traps found at the channel-dielectric interface.^37^ The hysteresis can be reduced by postcontact annealing. The output characteristics for a device annealed in air at 300 °C for 4 h are shown in Figure 1b.

**Figure 2:**
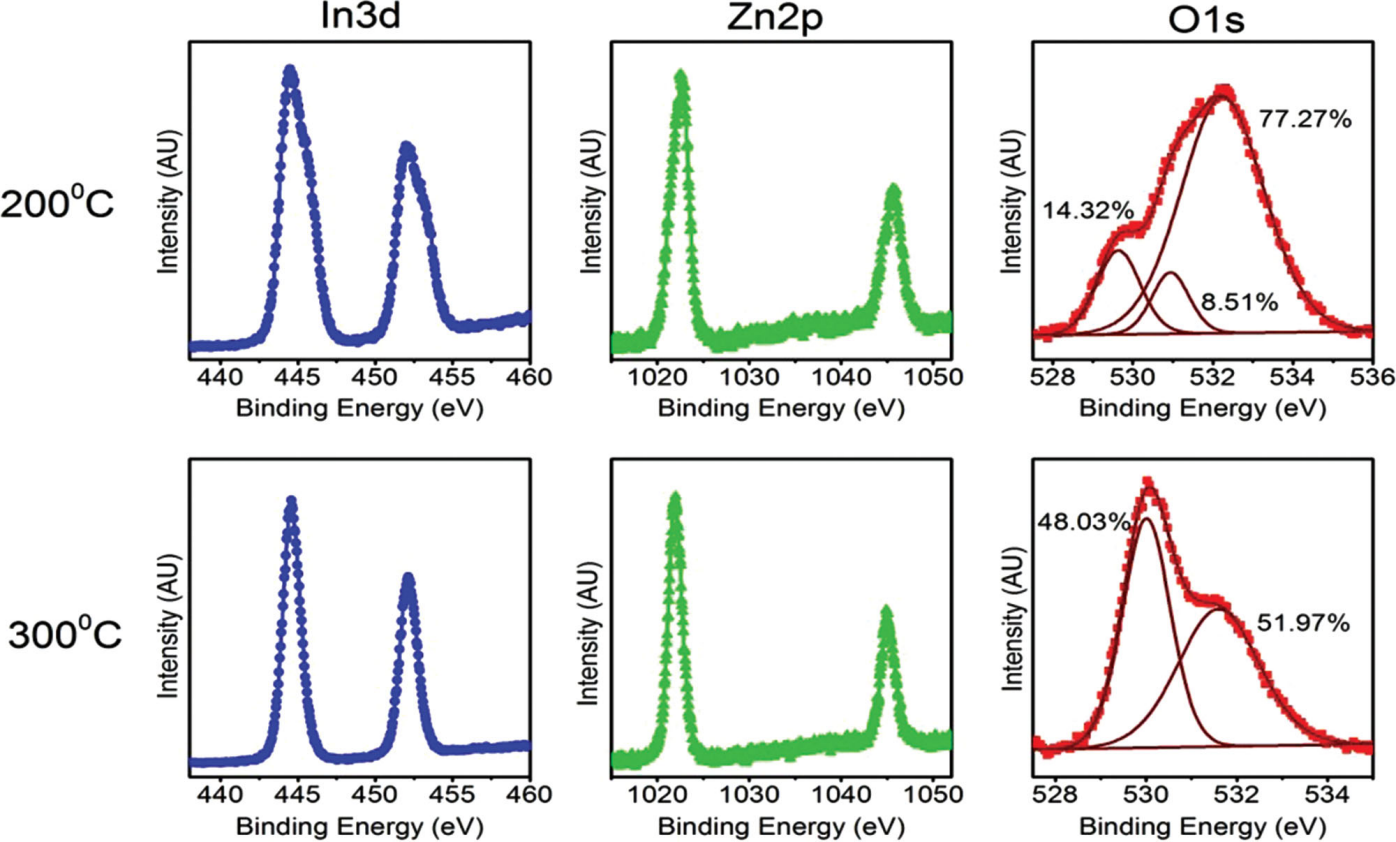
XPS data for IZO (In_2_O_3_:ZnO = 6:4) films annealed in air for 4 h at 200 °C (top) and 300 °C (bottom). Left: In 3*d* core level. Middle: Zn 2*p* core level. Right: Oxygen 1*s* core level resolved into Gaussians representing different oxygen binding environments: 1) OH species at a binding energy of ≈532 eV; 2) fully coordinated oxygen at ≈530 eV; and 3) oxygen vacancies at ≈531 eV (only resolved for the 200 °C sample). The XPS data were taken on the surface of the films.

In order to account for this dramatic decrease in performance with decreasing annealing temperature, we performed XPS analysis on films processed at different temperatures. XPS data for samples annealed at 300 and 200 °C are shown in **Figure** **2**. The XPS was performed on the surface of the samples since etching resulted in contributions to the XPS signal from the SiO_2_ surface since the films are only ≈10-nm thick. In all samples, the nitrogen contribution was at the limit of XPS resolution, suggesting that nitrogen species are quickly eliminated from the samples even at 200 °C in agreement with other reports.^34^ The indium and zinc peaks broaden with decreasing temperature suggesting the existence of different species. At high temperatures, the film is mainly composed of M–O–M species, whereas at low temperatures, the metal hydroxide precursor decomposition is not complete and there are still many M–OH species from the precursor. This results in a core level shift in both the indium and zinc peaks at low temperatures. This observation is more evident in the indium peaks suggesting that indium hydroxide species may convert into fully formed M–O–M bonds at higher temperatures than the zinc hydroxide species.^30^,^31^ The O1*s* peak is usually decomposed into three peaks representing three different environments: (1) OH species at high binding energies (2) fully coordinated oxygen at low binding energies (3) oxygen vacancies at intermediate binding energies. The OH peak contains both surface and bulk OH. The surface contribution of OH, although significant, should not be very different between samples since all of them have had the same air exposure before being measured. We can, therefore, still compare the contribution of bulk OH between samples. At 200 °C, the three peaks are clearly shown. It is observed that the film is dominated by OH species, which is clear evidence that the oxide skeleton has not fully formed. At this temperature, the film is a mixture of different hydroxide complexes and free hydrogen ions. The OH peak is significantly decreased at 300 °C compared with the sample annealed at 200 °C since through olation and oxolation, these species have evolved to fully coordinated oxygen as shown by the latter peak which has increased proportionally. The removal of precursor OH and their conversion to fully coordinated oxygen is likely to be the main reason for the large difference in performance between 200 and 300 °C.

From the O1*s* core level fit to three components, including an oxygen vacancy level, we find that at 200 °C, ≈8.5% of the oxygen atoms are bound to a metal atom that also has an oxygen vacancy attached to it. We note that the actual oxygen vacancy concentration is likely to be less than ≈8.5% since more than one oxygen atoms can feel the presence of the same vacancy. We could not resolve the oxygen vacancy peak in the sample annealed at 300 °C, since the spectrum is dominated by the OH and fully coordinated oxygen peaks and a good fit of the oxygen core level could already be obtained by assuming two peaks only. Instead, we used the composition of each individual element acquired from XPS and the fact that there are 1.5/1 oxygen atoms for each In/Zn atom to arrive at an alternative estimation of the oxygen content of the films. This calculation is shown in Table S1 (Supporting Information) and shows that the oxygen ratio is ≈7% lower than expected at 300 °C. It should be noted that to estimate this, the full OH peak, which contains both surface and bulk OH, has been taken into account. Since surface OH is not present in the bulk, this method underestimates the oxygen vacancies. This compositional analysis cannot be done on the 200 °C film since at this temperature, the film comprises still a significant fraction of metal hydroxide precursor species for which, the formula O/(1.5 In + Zn) for the oxygen ratio, which assumes that In_2_O_3_ and ZnO are fully formed, cannot be used. It is expected that some of the OH detected are surface OH and hence, that relatively vacancy concentration at 300 °C could be higher than the estimated 7%. In order to compare the oxygen vacancy concentration at different temperatures, we performed compositional analysis between samples annealed at 300, 350, and 400 °C (Table S1, Supporting Information). After the surface compositional analysis, we etched the samples in the XPS chamber and obtained a bulk scan. Using the surface and bulk scans we were able to verify that the OH surface species were similar between the samples and thus, the relative oxygen vacancy concentration between them can be compared. This study revealed that the variation between oxygen vacancies is not more than 3% in a temperature range of 100 C. This is in agreement with other observations, which found that oxygen vacancy concentration does not change much with temperature.^34^ It is, however, expected that as the samples are annealed at higher temperatures, some of the oxygen vacancies form shallow donor levels near the CBM rather than deep localized states near the VBM.^12^ This could provide a sufficient donor concentration to fill all acceptor states near the CBM and in this way, enhance electron transport.^38^–^41^ This could be a second reason why the mobility and on-current are enhanced with increasing annealing temperature. Figure S5 (Supporting Information) shows corroborating electrical data for the quaternary oxide IGZO for different gallium doping. The presence of gallium is known to suppress oxygen vacancies due to its strong bond with oxygen and hence, decrease the oxygen-induced shallow donor concentration.^9^,^12^,^42^,^43^ This decreases the charge-carrier mobility with increasing Ga doping as observed in Figure S5 (Supporting Information).

**Figure 3:**
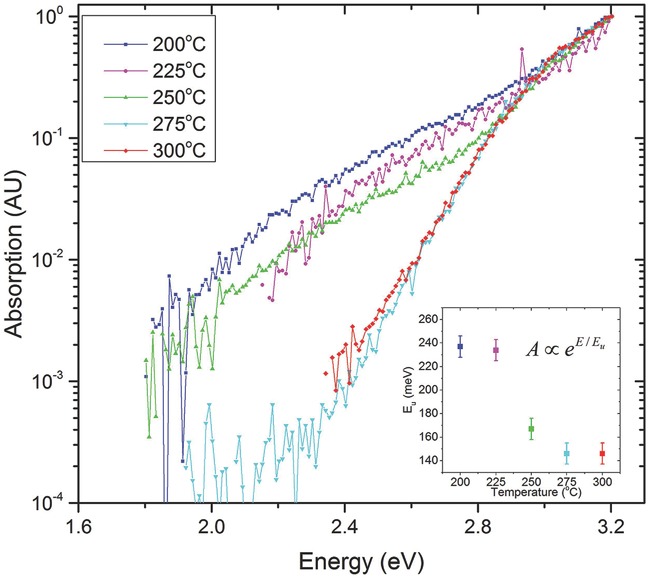
PDS data at different temperatures. The data are normalized with respect to the maximum absorption close to the bandgap energy ≈3.2 eV to eliminate any effects arising from different thicknesses between samples. The absorption within 450 meV of the bandgap energy is used to calculate an Urbach energy *E*_u_ (Figure S7, Supporting Information). This is a measure of the disorder near the VB and is of the order of 140–240 meV as shown in the inset.

We also performed PDS, which is a very sensitive optical absorption technique, to compare the density of states between samples annealed at different temperatures. A brief explanation of PDS and a diagram of the setup are shown in Figure S6 (Supporting Information).^44^,^45^ **Figure** **3** shows PDS data normalized with respect to the absorption at the bandgap energy (≈3.2 eV) in order to eliminate any effects originating from different film thicknesses between samples. Thinner samples can show a decreased absorption ≈3.2 eV but normalizing all samples eliminates any ambiguities. We can see that the sub-bandgap optical absorption due to trap states within the bandgap decreases with increasing temperature since the absorption at each energy is less. Above 250 °C, we detect a pronounced decrease of the deep sub-bandgap absorption around 2.4 eV, which is remarkably well correlated with the temperature at which the device performance becomes high and stable. The associated trap states could originate from the precursor which, as seen in our XPS data at low temperatures, has not yet decomposed. From the tail of the optical absorption closer to the bandgap, an Urbach energy can be extracted as the decay constant of an exponential fit of the PDS spectrum in the energy range from 2.8–3.2 eV (Figure S7, Supporting Information). The Urbach energies are in the range of 140–240 meV, which is relatively large compared with other amorphous semiconductors, such as amorphous silicon or organic semiconductors. There is a clear reduction of the Urbach energy for annealing temperatures ≥250 °C (inset of Figure 3). The clear correlation between PDS and FET data suggests that the changes in PDS spectrum upon annealing do at least partly reflect a reduction in the concentration of unoccupied electron trap levels below the CBM due to either removal or passivation of the associated defect or filling of the trap level by a compensating, hydrogen- or oxygen vacancy-induced donor level as opposed to a reduction in the occupied DOS near the VBM that are not expected to directly affect electron transport.

### 2.2.  Effect of Additional Vacuum Annealing

To investigate the nature of the defect states involved further, we now turn to studying the evolution of the electronic structure during an additional vacuum annealing step after the 4 h air anneal, which was recently reported^34^ to further enhance device performance. We first observe the change in TFT characteristics produced by introducing this vacuum annealing step and then we use spectroscopic techniques to investigate the effect of vacuum annealing on the electronic density of states.

#### 2.2.1. Electrical Characteristics

**Figure 4:**
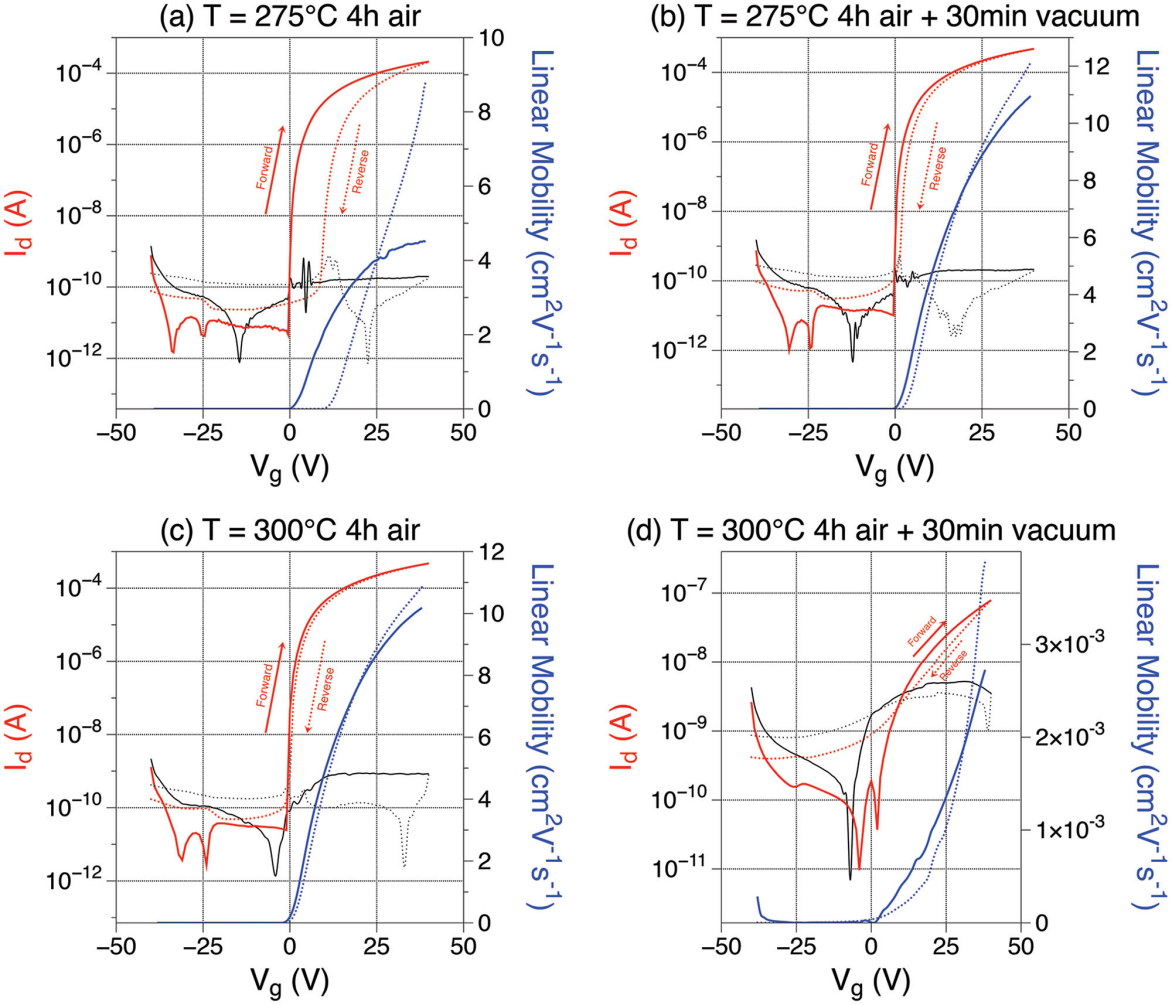
Red: drain current. Black: gate leakage current. Blue: linear mobility. Solid lines represent forward scan. Dotted lines represent reverse scan. Transfer curves for samples annealed at different temperatures in air with and without an additional vacuum annealing step (In_2_O_3_:ZnO = 6:4, W/L = 3000/300 μm, *V*_d_ = 5 V): Left: 4 h air anneal only; Right: 30 min additional vacuum annealing; Top: 275 °C; Bottom: 300 °C.

We used the same oxide ratio as before and fabricated bottom-gate TFTs using the same method. We added an additional half an hour vacuum annealing step after the 4 hof air anneal, at the same temperature as the air anneal. Devices with the extra vacuum anneal will be referred to as “with vacuum”, whereas only air annealed ones as “without vacuum” or “only air anneal”. **Figure** **4** shows devices annealed at 300 and 275 °C. At each temperature, a device with vacuum and one without vacuum annealing has been prepared. The mobility at 275 °C, increases from 4.5 to 11 cm^2^ V^−1^ s^−1^ by adding the vacuum annealing step. The hysteresis in the transfer characteristics decreases from 9.6 to 1.7 V. Additional vacuum annealing seems to be beneficial in this case, in agreement with other findings.^34^,^46^,^47^ We would expect to see the same improvement at 300 °C. Instead, surprisingly, the device at 300 °C with vacuum annealing stops working. This result is reproducible, which leads us to believe that, under certain circumstances, vacuum annealing introduces defect states that trap electrons and hinder charge transport. In the following, we aim to find experimental evidence for the nature of these defects using XPS, UPS, and PDS.

#### 2.2.2. Spectroscopy

**Figure 5:**
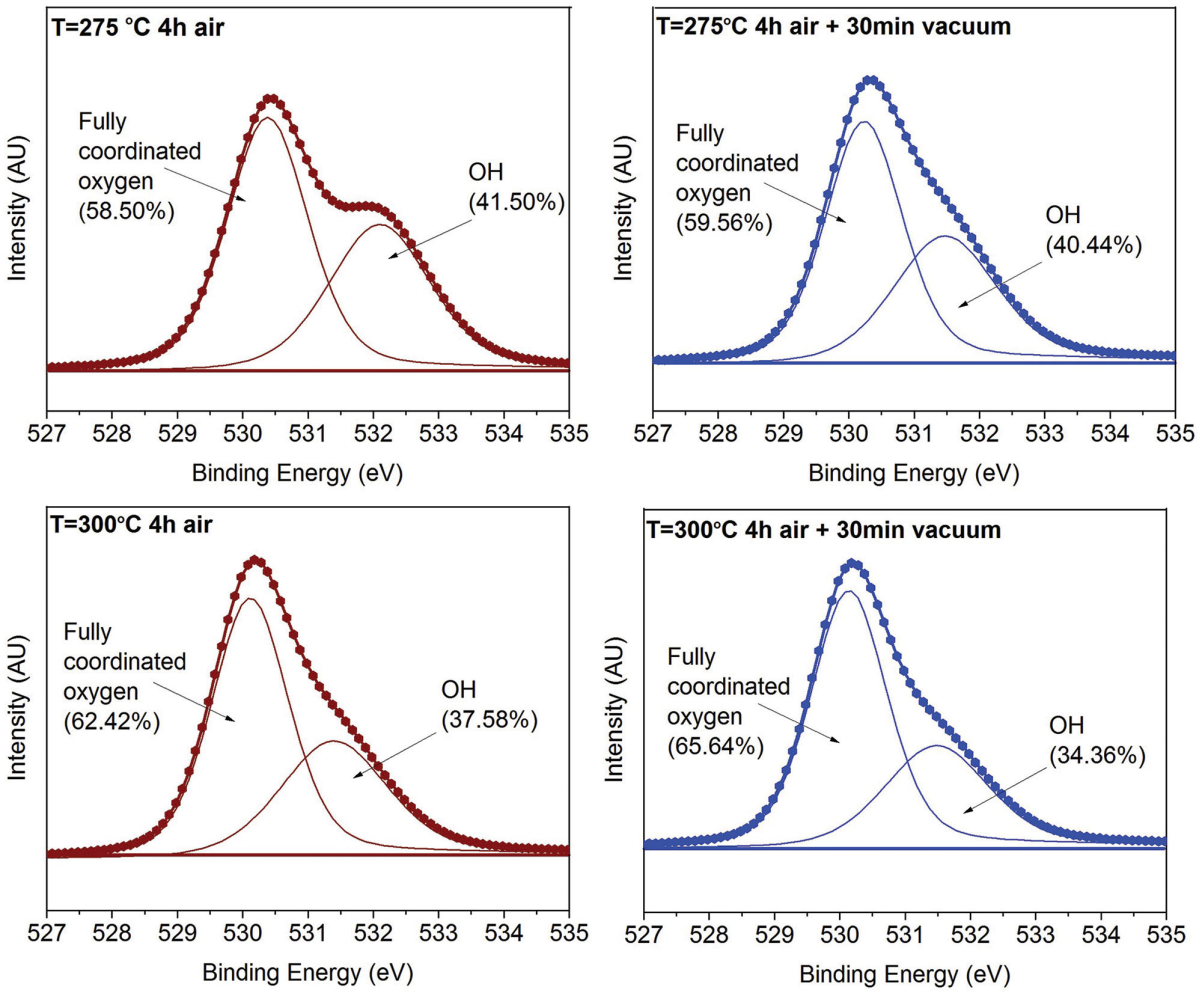
XPS O1*s* peak for samples annealed at different temperatures in air with and without an additional vacuum annealing step (In_2_O_3_:ZnO = 6:4): Left: 4 h air anneal only; Right: 30 min additional vacuum annealing; Top: 275 °C; Bottom: 300 °C. We can distinguish two oxygen binding environments: 1) OH species at ≈532 eV; and 2) fully coordinated oxygen at ≈530 eV.

**Figure** **5** shows XPS data for the four devices shown in Figure 4. The XPS data were acquired after etching of ≈80 s to remove the surface contamination. Figure S8 (Supporting Information) shows the oxygen 1*s* peak at different etching times. After 60 s of etching, we reach the bulk of the film (OH shoulder decreases significantly compared with surface and carbon completely disappears) and remain at it until 100 s. From this point onwards, we start getting contributions from the Si substrate as the shoulder at ≈531.7 eV increases and shifts to higher binding energies. We, therefore, present data at ≈80 s to make sure we have the minimum contribution from the surface contamination or Si substrate. Since the samples might have different thicknesses, we performed this analysis on each of them individually. It was found that ≈80 s of etching was the optimum for all four samples implying that the films had similar thicknesses. We therefore, present the data for this region. As before, nitrogen was absent from the bulk of our samples, suggesting that no nitrogen residuals remain at these temperatures. Nitrogen was only observed as a surface contaminant in one of our samples (Figure S9, Supporting Information).

The oxygen 1*s* peak reveals that vacuum annealing further removes M–OH species and boosts the oxide formation mainly at 300 °C. At 275 °C, only ≈1% of the remaining OH species are removed by the vacuum annealing, whereas at 300 °C, the OH peak decreases by a further ≈3%. At 300 °C, there are ≈37.5% M–OH remaining after the air anneal compared with ≈41.5% at 275 °C. This observation and the fact that after vacuum annealing the M–OH peak decreases by 3× more at 300 than at 275 °C, implies that there are now significantly less (≈6%) OH remaining after the vacuum annealing at 300 than at 275 °C. Furthermore, as shown in Figure S10 (Supporting Information), the OH peak continues to decrease in only air annealed samples at 350 °C, which continue to work nicely. The reduction of OH detected by XPS is thus, attributed to the precursor decomposition and would in principle be expected to boost device performance at 300 °C in vacuum as it does at 275 °C. This suggests that the reason for the device degradation after vacuum annealing at 300 °C is related to a defect that is not directly probed by core-level XPS.

The results reported so far can be explained consistently by assuming that during vacuum annealing hydrogen-induced, compensating shallow donor or passivating defects are eliminated from the film. This could be either due to removal of substitutional hydrogen *H*_o_ or interstitial hydrogen *H*_i_ dopant levels that act as compensating donors for acceptor states below the CBM. *H*_i_ has previously been found to decrease significantly after vacuum annealing at 350 °C,^22^ although *H*_i_ has also been reported to be unstable in annealed samples and may not be present in large amounts in our 300 °C films.^19^ Alternatively, hydrogen acting as passivation to a defect that would act as an electron accepting trap state, such as the zinc vacancy passivating defect H–II, may be eliminated from the films. Of course, it is also possible that a new, as yet unidentified defect is created under vacuum. However, our postulated, crucial beneficial role of hydrogen-induced defects is in agreement with other reports, which have found a significantly lower concentration of hydrogen atoms by XPS than by secondary ion mass spectroscopy.^48^

**Figure 6:**
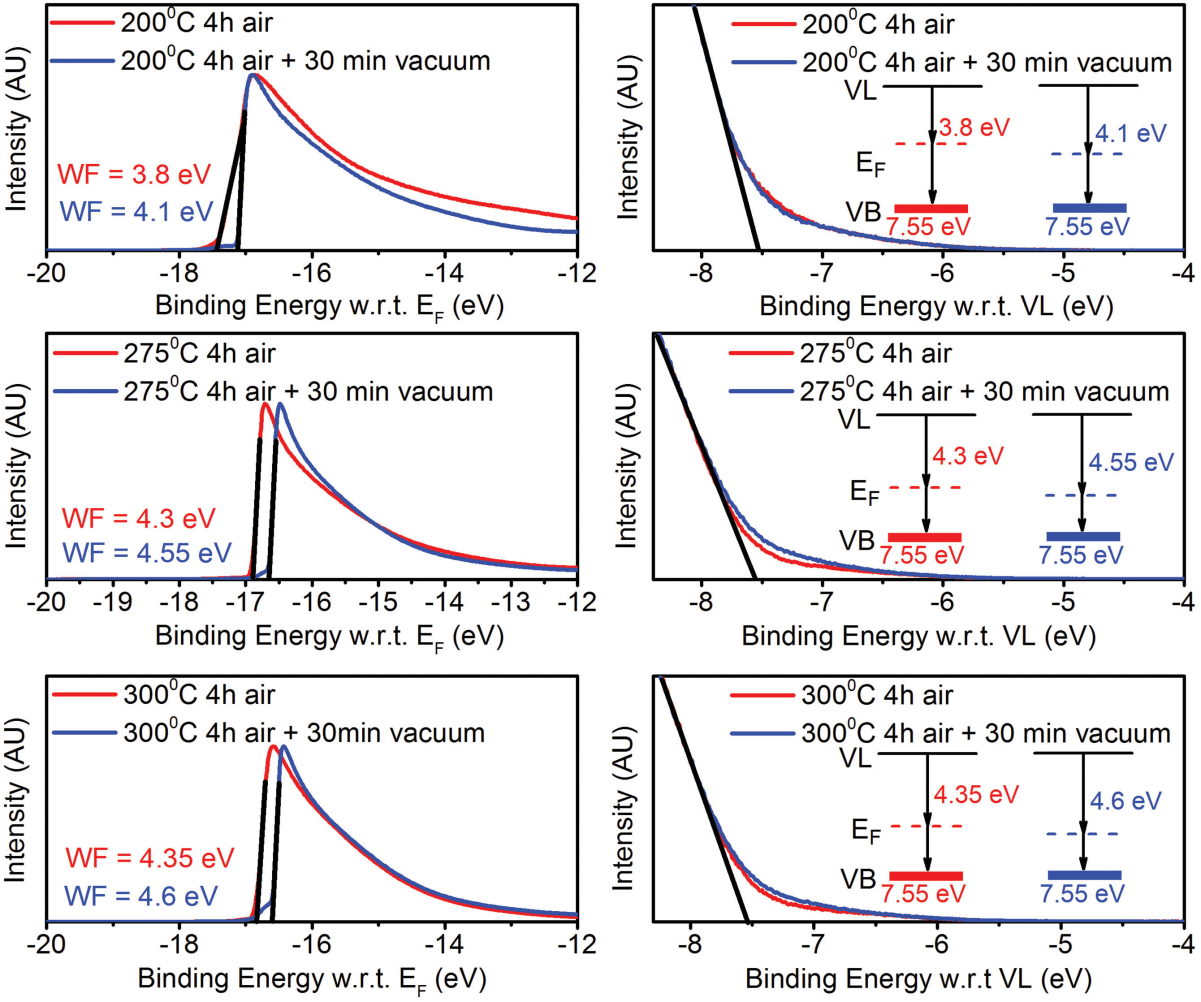
Ultraviolet photoemission spectra of samples annealed at 200, 275, and 300 °C for 4 h in air (red) and additional 30 min in vacuum (blue). Left: secondary photoemission onset determining the position of the Fermi level. Right: valence band region. (Inset: corresponding energy level diagrams). The photon energy is 21.2 eV. The work function was found by using the energy value at which the secondary photoemission begins (intersection of black lines with *x*-axis on the left diagrams) and adding to this the photon energy of 21.2 eV. The valence band edge energy was found by extrapolating the slope of the absorption (black line on the right diagrams) and reading the value of its intersection with the *x*-axis.

To investigate the nature of these defects further, we have extracted energy level diagrams from the UPS work function and valence band edge measurements shown in **Figure** **6**. In these measurements, we were careful to avoid charging effects that can induce errors in the extracted energy levels. First, very thin films were used for the measurements (<10 nm), deposited on conductive substrates and carefully connected to the sample holder. Second, the measurements were performed with two different biases (–5 and –10 V) to ensure that no charging is taking place. Finally, the oxide films used in this study are significantly more conductive than other oxide films such as TiO_2_, the energy levels of which are routinely measured by UPS. The work function was found by using the energy value at which, the secondary photoemission begins (intersection of black lines with *x*-axis on the left diagrams of Figure 6) and adding to this the photon energy of 21.2 eV. The valence band edge energy was found by extrapolating the slope of the photoemission onset (black line on the right diagrams of Figure 6) and reading the value of its intersection with the *x*-axis. The Fermi level *E_f_*, in the 275 and 300 °C, only air annealed samples lies very close to the conduction band (since the bandgap is ≈3.2eV (Figure S11, Supporting Information)), which is consistent with the reports on sputtered oxides.^9^,^10^ This is what gives these high bandgap materials their n-type semiconducting properties. The UPS data for the only air annealed sample at 200 °C show an even lower work function compared with 275 and 300 °C, indicating that already at 200 °C a fraction of the large concentration of M–OH precursor species that are present in the film, as evident in XPS data (Figure 2), may act as dopants. Indeed, we know that any hydrogen in the film at these temperatures is either OH from the precursor or from interstitial hydrogen *H*_i_, which could be stable at low temperatures and generate electrons, which move the Fermi level up. Merely from a Fermi level perspective, one may expect that the devices at 200 °C would behave better since the H-induced dopant species push *E_f_* closer to the conduction band. However, as seen from our XPS data, the dominant effect on electron transport is that the decomposition of the precursor remains incomplete at the temperatures, which is likely to lead to significant disorder in the density of states near the CBM, as seen clearly also in the PDS measurements above, and to severely inhibit charge transport and carrier mobility.

Upon vacuum annealing, the Fermi level for the 275 and 300 °C samples moves ≈250 meV away from the conduction band compared with the corresponding only air annealed samples. The lowering of the Fermi level suggests that the density of shallow dopant levels has been reduced by annealing. While the replacement of M–OH species from unreacted precursor molecules with fully coordinated M–O–M bonds during the air-anneal is generally beneficial to transport, the removal of *H*_i_ or *H*_o_ species that are acting as H-induced shallow dopant states that are available to fill any acceptor states near the CBM is expected to lower the Fermi level and degrade transport.^12^ This observation applies to both 275 and 300 °C samples. At 275 °C, however, the thermal energy is less than 300 °C and the diffusion of hydrogen from the buried oxide channel to the surface is likely to be slower. We therefore hypothesize that after the 275 °C annealing, some of the H-induced donor states remain present at the buried interface, while they have already been removed at the surface. UPS probes the surface of the films and does not reveal what is happening at the interface. In this way we can explain that, although the UPS measurements show evidence for an increase in workfunction upon vacuum annealing in both samples, the 275 °C vacuum annealed TFTs keep functioning well, while at 300 °C they cease operating. The removal of the donor states at the interface at 300 °C leaves any acceptor-like states below the CBM unoccupied and allows them to act as electron traps. At 275 °C, enough H-induced donors remain present at the buried interface to fill these traps and the TFT improves, because of the beneficial effect of converting residual unconverted M–OH precursor species to fully coordinated oxide. This explanation is fully consistent with our observation (Figure S12, Supporting Information) that the TFTs stop working even in vacuum-annealed 275 °C samples, when annealed for longer (one hour instead of 30 min). This can be explained by a sufficient time for diffusion/removal of the residual hydrogen from the buried interface. In addition, a TFT annealed in air at 275 °C for 4 h, and then placed in vacuum at 300 °C for 15 min stops working (Figure S12, Supporting Information). This indicates that at 300 °C, the thermal energy is large enough to make hydrogen diffusion, from the interface to the surface, a very fast process. Thus, 275 °C is close to the limiting temperature at which, annealing in vacuum can take place, provided that the annealing time is kept short (≈30 min).

The nature of the electron trapping acceptor states near the CBM, which manifest themselves in the device characteristics, upon elimination of hydrogen-related donor species during vacuum annealing is unclear at present. They could be the states at ≈0.8 eV below the CBM that were identified in refs. 9,10, which may then be compensated by the hydrogen donor species. However, they might also be of a different origin in our solution-processed films. They could be related to Zn vacancies *V*_Zn_, 13 which can be passivated by hydrogen 22,24 or they could involve the elimination of hydrogen from residual hydroxide species that then become able to trap electrons. This could be seen as an analogy to the dangling bond formation mechanism in covalently bonded hydrogenated amorphous silicon.^49^ Further experimental and theoretical work is required to identify the nature of these electron trapping acceptor states near the CBM.

From our UPS data, we also see that the 300 °C sample has a slightly lower *E_f_* than the 275 °C one by ≈50 meV. If we consider that the CB tail is only 80–150 meV,^9^,^10^ this 50 meV shift could make a significant contribution to the TFT performance, since it can move the Fermi level through the band tail near the CBM and leave any trap states unfilled. This would, however, probably manifest itself as a positive shift in the onset voltage of the TFT, which we do not observe. In addition, we note from the UPS data near the VB onset that vacuum annealing also increases incrementally the tail state density near the VBM. The increase in VB tail states is not expected to strongly affect the TFT properties since these states are very deep and therefore, filled.^9^,^10^ The disorder produced by them could deteriorate the performance and reduce the mobility but not by three orders of magnitude as observed at 300 °C. Furthermore, these states are present at both 275 and 300 °C.

**Figure 7:**
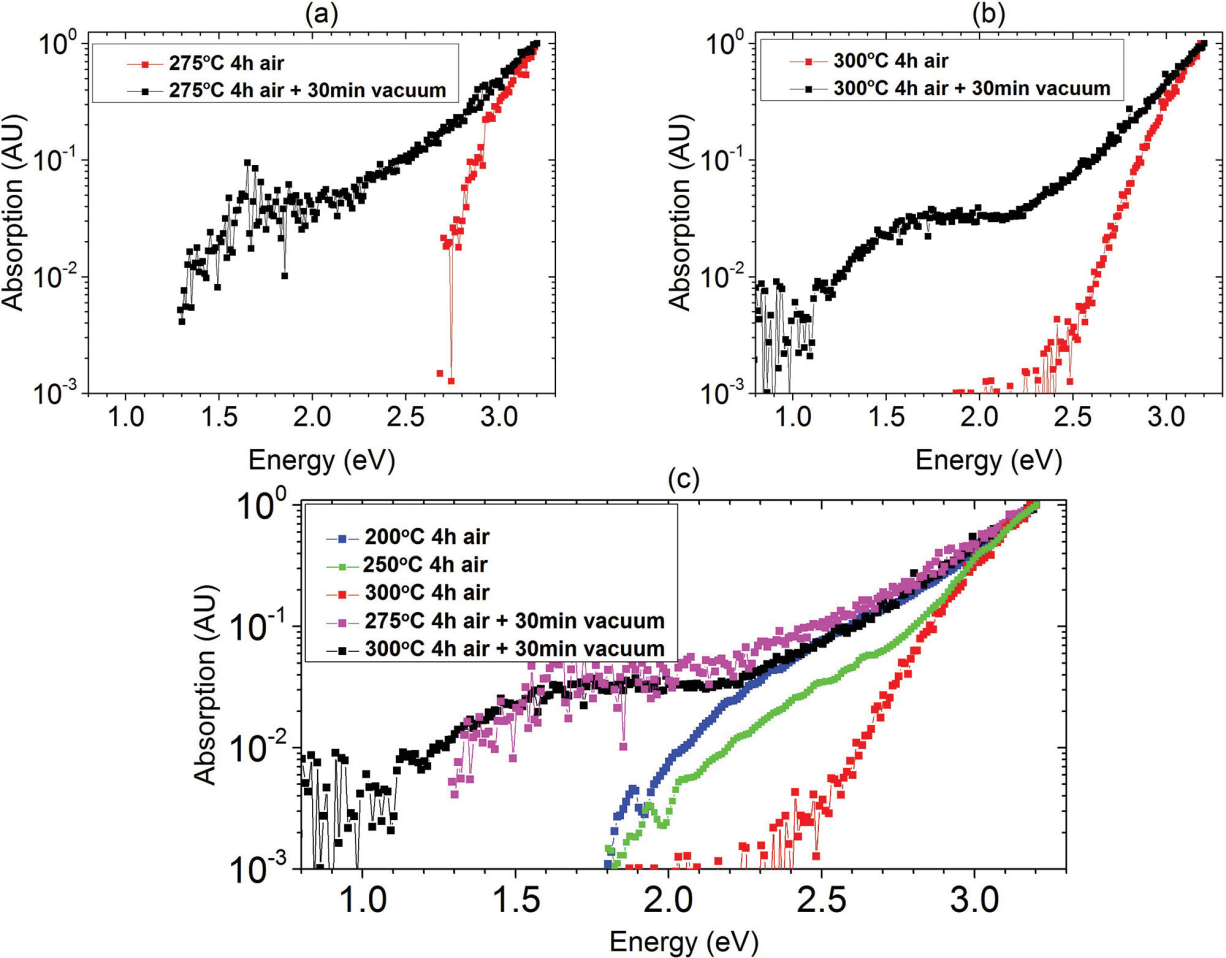
PDS spectra. The data are normalized with respect to the maximum absorption close to the band gap energy ≈3.2 eV to eliminate any effects arising from different thicknesses between samples. a,b) Samples annealed in air for 4 h (red) and samples with additional 30 min of vacuum annealing (black) at 275 °C a) and 300 °C b); c) Comparison between samples with 30 min additional vacuum annealing at 275 °C (pink) and 300 °C (black) with only air annealed samples at 200 (blue), 250 (green), and 300 °C (red).

In order to observe optically any sub-bandgap states, we performed further PDS measurements. The PDS data are shown in **Figure** **7**. As earlier, we normalized the data to the maximum absorption near the bandgap energy (≈3.2 eV). This ensures that any effects due to different film thicknesses are eliminated. It is clearly seen that the vacuum annealed samples exhibit a significantly higher sub-bandgap absorption, which extends down to ≈1.5 eV before reaching the noise level of the experiment (≈10^−4^), whereas the only air annealed samples exhibit a much cleaner bandgap with a sharp onset of absorption around 2.5 eV (Figure 7a,b). The optical absorption coefficient reflects the joint density of states of occupied and empty energy levels near the VBM and CBM, respectively and it is difficult to extract from PDS alone where in the bandgap these states are located. However, the correlation between electrical data and PDS results strongly suggests that the band of sub-bandgap transitions appearing in PDS in the samples with vacuum-annealing are transitions from states at or close to the VBM to the now unoccupied acceptor states below the CBM and may be a sensitive probe of any electron trap states below the CBM. Since PDS is a bulk technique, the signal is similar at 275 and 300 °C (Figure 7c), even though the acceptor states at the buried channel at 275 °C may still be occupied by electrons from H-induced dopant, as discussed above. Figure 7c also shows PDS data for only air annealed devices at 300, 250, and 200 °C. It is seen that the absorption for the only air annealed samples at 200 °C follows that of the samples with additional vacuum annealing up to ≈2.3 eV. The Urbach energy of the vacuum annealed samples is similar to the only air annealed sample at 200 °C (Figure S13, Supporting Information). This could indicate that some of the defect states existing in the low temperature (200 °C) air-annealed samples return upon vacuum annealing, i.e., that air annealing at temperature of 250–300 °C does not in fact eliminate these defects, but merely fills them with electrons from thermally activated shallow donor states.

## 3. Discussion

A consistent explanation of our observations can be obtained by assuming that the reappearance of sub-bandgap transitions in PDS reflects at least partly transitions related to the removal of *H*_o_, which leaves the metal atoms’ dangling bonds uncompensated. These could form the a_1_ state close to the VBM, which could generate optical transitions at energies close to the bandgap energy. This state would also exist at low temperatures (200 °C) in air, since *H*_o_ is not likely to have formed, yet at these temperatures because its formation energy is higher than *H*_i_.^19^ As seen from XPS at 200 °C, most of the hydrogen is in the precursor state or exists as an interstitial since *H*_i_ is expected to be most stable. The a_1_ site is located deep near the VB and can contribute to the absorption since it is occupied by two electrons. As the temperature is increased, *H*_i_ is expected to become increasingly unstable and the hydrogen released from the precursor decomposition may be assumed to mostly form *H*_o_. The substitutional hydrogen removes the a_1_ state from its previous position and creates a fully occupied state in the VB as well as donates an electron to the CB, which is then also available to compensate any electron acceptor states below the CBM. The associated a_1_ absorption and any absorptions due to unfilled acceptor states are thus removed at higher air annealing temperatures. When *H*_o_ is removed in vacuum, a_1_ forms again as a deep level near the VBM and the acceptor states below the CBM become unfilled again. Both of these effects can contribute to sub-bandgap optical absorption at energies near E_g_. This can also be seen from our UPS data near the valence band edge that show an increased density of occupied states close to VBM. Below 2.3 eV, the PDS absorption for the 200 °C, only air annealed sample is weak compared with that of the samples with additional vacuum annealing. This is consistent with the UPS results, which show that at 200 °C, any deep acceptor levels below the CBM are expected to be filled by electrons from shallow, *H*_o_ or *H*_i_ donors. We also emphasize that the observed changes in PDS spectra after vacuum annealing are permanent, films stored in air for 6 months after annealing show exactly the same trend as when measured directly after annealing (Figure S14, Supporting Information).

We note that vacuum annealing may also result in a small decrease of oxygen vacancy concentration. Our XPS composition analysis (Table S2, Supporting Information) suggests that oxygen vacancies are suppressed by 4% and 3% in vacuum annealed samples at 275 and 300 °C, respectively, compared with the only air-annealed samples. The vacuum-annealed samples and the only air-annealed one at 300 °C had almost no surface OH and could be compared. The only air annealed sample at 275 °C had slightly more surface OH and the oxygen vacancy concentration is expected to be higher than the estimated one. From the UPS valence band edge measurements in Figure 5, we know that the density of occupied states near the VBM increases slightly, rather than decreases, for samples with vacuum annealing. This implies that reduction in oxygen vacancy concentration during vacuum annealing is not due to elimination of configurations leading to filled states near the VBM, but rather of configurations that result in shallow donor levels. The removal of oxygen vacancy donors would have a similar effect on the electrical performance as the removal of the hydrogen donors: any acceptor states near the CBM would remain unfilled and would act as electron traps. However, we believe that the dominant effect appears to be the reduction in the concentration of hydrogen induced donor states, because the device degradation upon vacuum annealing is more pronounced in the 300 °C samples, which have a significantly (6%–7%) higher oxygen vacancy concentration than the corresponding 275 °C samples. It is of course possible that there exist other defects near the CBM, which we are not able to detect. An example of such a defect could be a zinc interstitial, which was previously found to form a shallow donor at ≈30 meV below the CBM.^50^ It is possible that a local structural rearrangement in vacuum, could eliminate these donors and leave more of the acceptor states unfilled.

The complexity of hydrogen-related defects together with the existence of many native defects in oxides, makes it difficult to directly observe their fingerprint. In order to understand the defects causing the poor device performance at low annealing temperature, and the degradation of the device upon vacuum annealing, we combined a variety of highly sensitive spectroscopic techniques that have enabled us to eliminate certain hypotheses and favor candidate defects. First, our XPS and electrical results indicate that our films do not comprise extrinsic impurities in significant concentrations; therefore, the defect that is apparent in PDS and so clearly correlated with the electrical data has to be associated with a native one. Our XPS and UPS results suggest a reduction in the concentration of shallow donor oxygen vacancy sites upon vacuum annealing. XPS also shows that the vacuum annealed sample at 300 °C that stops working well has more oxygen missing than the corresponding sample at 275 °C that still works nicely. Since unpassivated oxygen vacancies can either form beneficial shallow donor levels or fully occupied, inactive deep levels, they are unlikely to be responsible directly for the device degradation. Zinc interstitials, which form ≈30 meV below the conduction band have the highest formation energy of all native defects in n-type oxides and are unlikely to be present at high concentrations in O-rich oxides.^13^ Their concentration was, however, reported to increase rapidly with oxygen vacancy concentration.^51^ The interaction between an oxygen vacancy site and a zinc interstitial lowers the overall energy of the system, and hence, zinc interstitials are favorable in O-deficient oxides. Although our films are deposited and annealed in oxygen-rich conditions, our XPS results show that some of the oxygen is removed after annealing. If this is enough to induce a large number of zinc interstitials, the created donors could act as compensating agents in a similar manner to interstitial hydrogen. A structural rearrangement in vacuum could eliminate the zinc interstitials and leave any trap states unfilled. Our observation that the devices with additional vacuum annealing for 30 min at 300 °C and for o1 h at 275 °C degrade, whereas the one annealed for 30 min at 275 °C continues to work well, point toward a diffusion mechanism. The fact that UPS and PDS measurements are similar between the 30 min 300 °C and the 30 min 275 °C devices implies that the change leading to the degradation of the device only occurs at the interface, which further supports the diffusion hypotheses. If the removal of zinc interstitials was the main reason for the device degradation, we would therefore, expect this species to diffuse out of the vacuum annealed films. Our XPS results (Table S2, Supporting Information) do not show any reduction in the zinc concentration in vacuum-annealed samples compared with only air-annealed ones; if anything there is evidence for a small increase in zinc concentration upon vacuum annealing. In addition, the samples annealed at 300 °C in vacuum exhibit a smaller oxygen ratio, i.e., a larger concentration of oxygen vacancies, than the samples annealed at 275 °C in vacuum. Both observations are not consistent with a model in which a loss of Zn interstitials stabilized in the presence of oxygen vacancies as claimed in a previous study^51^ is responsible for the observed device degradation upon vacuum annealing.

We have therefore focused in our discussion of the results mainly on hydrogen-related defects. The removal of hydrogen-induced defects acting either as passivating agent for an electron accepting defect, such as H–II for *V*_Zn_, or as *H*_i_ or *H*_o_ compensating donor for any electron trap/acceptor states below the CBM, which leaves these states unoccupied and free to trap electrons provides the most plausible and consistent model for explaining the observed results. H–II has previously been found to be stable up to vacuum annealing temperatures of ≈550 °C and is therefore, not likely to be responsible for the observed device degradation in vacuum. In addition, the zinc vacancy is known to form a partially occupied state very close to the valence band and the removal of hydrogen passivating this defect may not be expected to create optical absorption so far below the bandgap as evident in the PDS results. We therefore conclude that the elimination of hydrogen as a compensating donor defect provides the most consistent explanation of our results. Interstitial hydrogen is a possible candidate since it was found to anneal out at ≈350 °C in vacuum. The absorption up to ≈2.3 eV in PDS was similar between only air annealed samples at 200 °C and vacuum annealed samples. The 200 °C only air annealed samples are expected to have many *H*_i_ species and hence the recurring disorder after vacuum annealing is not likely to be related to the removal of *H*_i_. Furthermore, *H*_i_ was found to be unstable in higher temperature annealed samples, where *H*_o_ is expected to be the dominant donor species.^19^ These observations make *H*_o_ to be the most likely candidate responsible for the device degradation at 300 °C in vacuum. Removing hydrogen from a vacancy site could leave behind uncompensated metal cation dangling bonds, which form an occupied a_1_ site close to the VB. This could show up in the PDS at an energy close to *E*_g_. *H*_o_ is not likely to form at high concentrations in the only air-annealed samples at 200 °C since at this low temperature, *H*_i_ is more stable; hence the a_1_ transition can also be seen in these samples. This can explain why the PDS absorption near *E*_g_ is similar for vacuum-annealed samples and only air-annealed samples at 200 °C. Removal of *H*_o_ donor species would also leave any acceptor states below the CM unfilled leading to the electron trapping and device degradation and contributing further to the sub-bandgap PDS optical absorption observed after vacuum annealing. Within this framework, the PDS absorption band below 1.8–2eV, that is not present after 200 °C air annealing, could be due to transitions between occupied states at or near the VBM and unfilled acceptor states below the CBM. These would not be expected to be observed in the 200 °C air annealing samples due to the high, potentially *H*_i_-induced doping concentration of these films.

Finally, we would like to comment on the remarkable behavior of the FET turn-on characteristics. As seen from our data, the onset voltage of our TFTs on the forward scan from negative to positive gate voltages remains remarkably close to zero even for devices annealed at 235 °C, only the hysteresis between forward and reverse scan depends on annealing temperature and conditions (Figures 1 and 4). This behavior can also be explained consistently within the proposed model. The free electron that is released by a *H*_o_ species leaves the oxygen vacancy in a +1 positive charge state, but when compensating a nearby deep electron acceptor state below, the CBM renders the latter negatively charged. Since no net charge is generated, the compensation is not expected to influence the threshold voltage of the transistor. When a gate-voltage is applied, the electron accumulation layer begins to form near *V*_g_ = 0 V, but the accumulated electrons equilibrate with any shallow trap states near the CBM that have not been filled by *H*_o_-induced compensation and this is the cause of the observed hysteresis. As reported elsewhere, the presence of uncompensated deep acceptor states influences *V*_on_ while shallow interface trap states mainly manifest themselves as hysteresis. 49 This means that the hydrogen released from the precursor decomposition has fully compensated any acceptor states even at 235 °C. The improvement of the device upon further increasing the temperature is likely to originate from a reduction in shallow trap state concentration, possibly associated with complete elimination of the precursor. Annealing at higher than 300 °C starts to produce a negative shift in *V*_on_ (Figure S15, Supporting Information) since all deep acceptor states are compensated, most shallow interface trap states have been eliminated and now some of the electrons donated by hydrogen donors can become free electrons in the CB that need to be depleted before the transistor turns off.

## 4. Conclusion

Our work provides important insight into the electronic structure of low-temperature, solution-processed amorphous MOS, and the defect states that limit the minimum achievable processing temperature for thermally annealed samples to >200 °C. We have demonstrated that the conversion from the water soluble metal hydroxide precursor to the fully coordinated oxide is incomplete at a low temperature of 200 °C as evident in the chemical shifts of the indium, zinc, and oxygen 1s XPS core levels. We produced good working Indium–Zinc–Oxide TFTs with a mobility of 7 cm^2^ V^−1^ s^−1^ by air annealing for 4 h at the low temperature of 250 °C. An additional short vacuum annealing step at a temperature not higher than 275 °C is beneficial to convert any hydroxide precursor species remaining after air annealing, in agreement with other reports.^34^ However, our combined XPS, UPS, and PDS study suggests that the removal of a hydrogen-related donor species that occurs during vacuum annealing is also associated with an increase in the work function, which leaves a significant concentration of deep acceptor states below the CBM unfilled and leads to dramatic device degradation when the vacuum annealing treatment is performed for too long or at too high temperatures. The maximum temperature at which, the additional vacuum annealing step can be performed to produce a good working TFT is, thus, 275 °C provided that the annealing time is kept short (≈30 min). This ensures that hydrogen does not have enough time to diffuse from the buried channel to the surface and hence, that hydrogen donor levels are still present near the dielectric-oxide interface, where the TFT channel is located. Our results demonstrate clearly that the electronic structure of low temperature, solution-processed amorphous MOS is complex and that a good device performance can only be achieved through the compensation of a significant density of deep acceptor states below the CBM by shallow hydrogen-induced donor levels. Our study provides some of the necessary scientific understanding of the electronic structure of these materials that is likely to be required in the future to invent novel processing methods or precursor routes to overcome the process temperature limitation around 200 °C that most common oxide precursor routes currently suffer from and that prevents the use of these materials for printed electronics applications on low-cost plastic substrates.

## 5. Experimental Section

The TFTs were made on a bottom gate p-doped Si substrate with a thermally grown 100 nm SiO_2_ dielectric layer on top. The IZO solution was made by mixing indium nitrate hydrate from Sigma-Aldrich and zinc nitrate hexahydrate from Alfa Aesar, so that In_2_O_3_:ZnO = 6:4. A molar concentration of 0.15 m was achieved by adding 10 mL of DI water. The solution is stirred overnight and can be used for more than 3 months. In the case of IGZO, a gallium nitrate hydrate precursor from Sigma-Aldrich was used. The solution was filtered through a 13 mm diameter, 0.2 μm membrane PTFE syringe filter before spin-coating. The solution was then spin-coated at 5000 rpm for 30 s and directly placed on a preheated hotplate at 150 °C for 30 s. The sample was then directly transferred to a hotplate in air and annealed for 4 h. The hotplate was prestabilized at the required temperature before the sample was placed on it. An MTI furnace was used for the extra vacuum annealing step. This was also prestabilized at the required temperature before the sample was transferred in it. The sample was first annealed in air for 4 h, then left to cool down for 1 h and subsequently, transferred to the preheated vacuum furnace. After the vacuum anneal, the sample was exposed to air and left to cool down before being removed from the furnace. 50 nm tungsten source-drain contacts were sputter-coated through a shadow mask. The TFTs were patterned by etching in 3% HCl for approximately 1 min. They were encapsulated by spin-coating a layer of CYTOP on top of the contacts. More details about the fabrication procedures can be found in Figure S1 (Supporting Information). The electrical characteristics were measured on a low-noise probe station in air using an Agilent parameter analyzer 4156C controlled by a Labview programme. The XPS/UPS samples were fabricated by spin-coating the oxide on a conductive Si substrate with native thermal oxide and then annealing as for the TFTs. In the case of vacuum annealing, samples were re-exposed to air for the same time as only-air annealed samples before XPS/UPS measurements. The PDS samples were spin-coated on a 11 mm quartz spectrosil and annealed as before. The absorbance was measured in a spectrophotometer that spans from UV to visible wavelengths.
