# Supplementary material for: Electronic Structure of Low-Temperature Solution-Processed Amorphous Metal Oxide Semiconductors for Thin-Film Transistor Applications
Source: Adv Funct Mater. 2015 Feb 18;25(12):1873–85. doi: 10.1002/adfm.201404375 (PMC4503976; doi:10.1002/adfm.201404375)
Supplement: Supplementary file 1 [file adfm0025-1873-sd1.pdf]

Copyright WILEY-VCH Verlag GmbH & Co. KGaA, 69469 Weinheim, Germany, 2015.

ADVANCED FUNCTIONAL MATERIALS

Supporting Information

for *Adv. Funct. Mater.,* DOI: 10.1002/adfm.201404375

Electronic Structure of Low-Temperature Solution-Processed Amorphous Metal Oxide Semiconductors for Thin-Film Transistor Applications

*Josephine Socratous, Kulbinder K. Banger, Yana Vaynzof, Aditya Sadhanala, Adam D. Brown, Alessandro Sepe, Ullrich Steiner, and Henning Sirringhaus**

Supporting Information

Electronic structure of low-temperature solution-processed amorphous metal oxide semiconductors for thin film transistor applications.

*Josephine Socratous, Kulbinder Banger, Yana Vaynzof, Aditya Sadhanala, Adam Brown, Alessandro Sepe, Ullrich Steiner and Henning Sirringhaus**

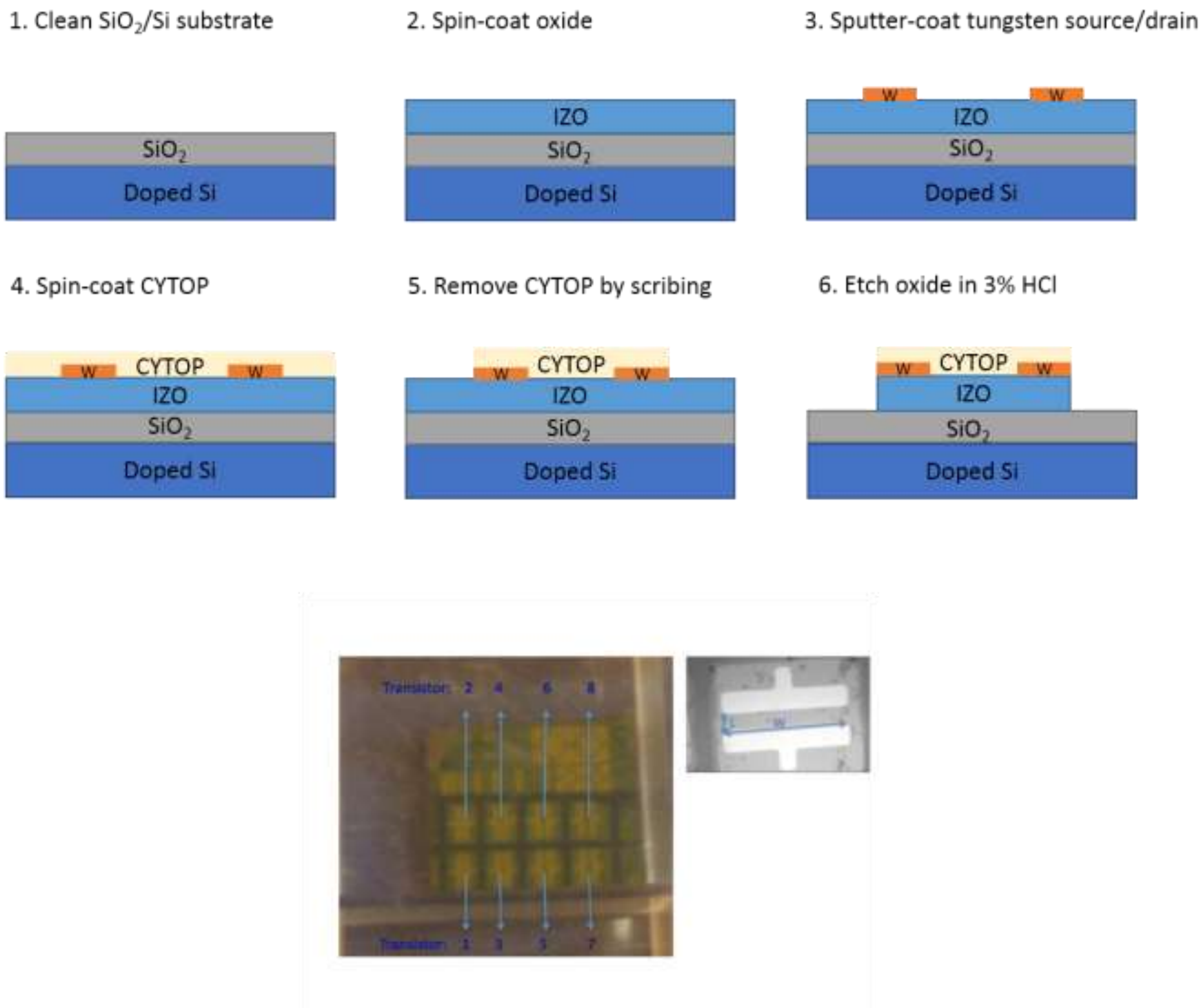

Figure S1. Thin-film transistors were fabricated by processes 1-6. A p-doped silicon substrate with 100nm thermally grown SiO_2 is cleaned via sonication in DI water, acetone and IPA. The oxide is then spin-coated from a solution using a syringe and a 13mm diameter 0.2µm PTFE syringe filter. The spinning speed is 5000rpm for 30s. After spin-coating the sample is soft-baked on a preheated hotplate at 150°C for 30s. It is then annealed in air for four hours. In the case of vacuum annealing the sample is further annealed in a vacuum furnace after the air anneal. After all annealing is finished the sample is left to cool down before 50nm of tungsten is sputtered as source/drain electrodes through a shadow mask. The

sample is then directly encapsulated by spin-coating a layer of CYTOP. The CYTOP is subsequently patterned by mechanical scribing and finally the oxide is patterned by etching in 3% water diluted hydrochloric acid. Bottom: Surface of our bottom-gate, top-contact device. Transistors are numbered. L=100µm for 1-2, 200µm for 3-4, 300µm for 5-6 and 400µm for 7-8.
W =3000µm for all transistors. The oxide semiconductor is etched in 3% HCl in the regions between the transistors, thus isolating them from each other. Contact to the gate is achieved by scribing a corner on the substrate. Inset shows a zoomed-in photograph of an individual transistor with the oxide around it removed.

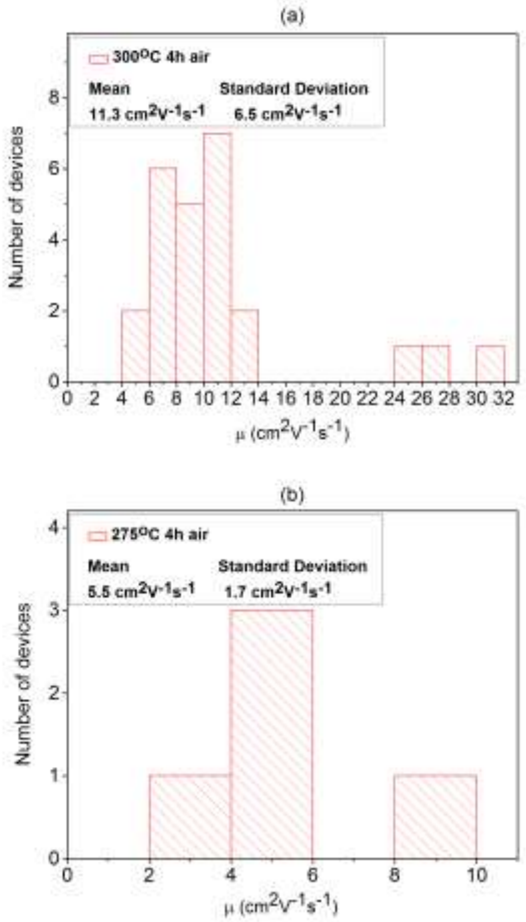

Figure S2. Histogram showing the spread in mobility for devices annealed in air for four hours at the same temperature. The devices were prepared at different periods. For each temperature the mean and standard deviation are also shown. (a) 300°C (b) 275°C.

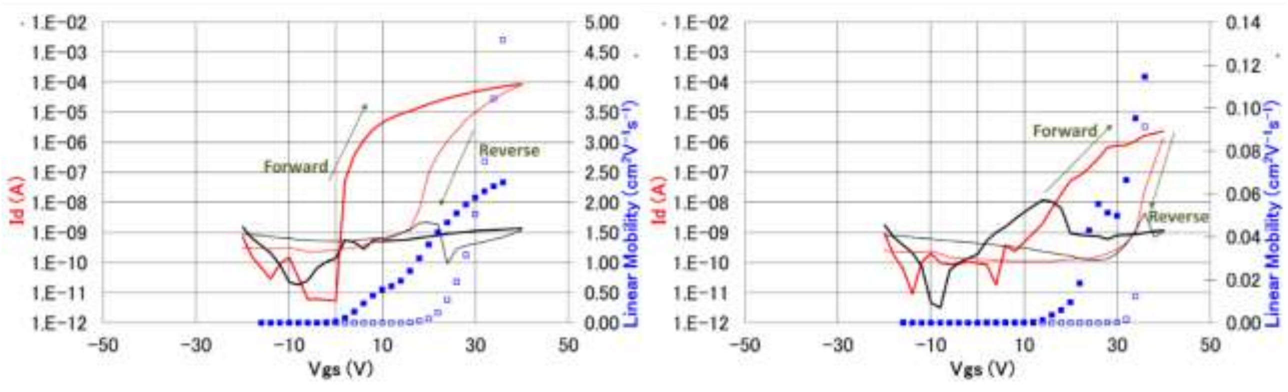

Figure S3. Red: Drain Current. Black: Gate leakage current. Blue: Linear Mobility. Hysteresis is clockwise as indicated by the arrows. Left: Device annealed for four hours in air at 235°C. Right: Device annealed for four hours in air at 215°C. At 235°C the TFT is still working but with a low mobility of ~$2.5 cm^2V^{-1}s^{-1}$ and large hysteresis. At 215°C the device behaves very poorly but TFT behaviour could still be observed.

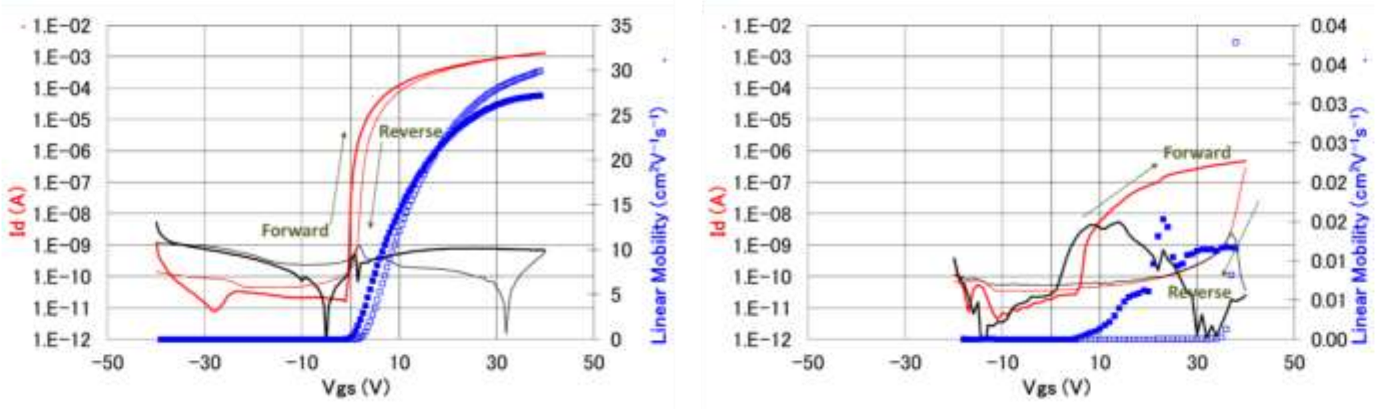

Figure S4. Red: Drain Current. Black: Gate leakage current. Blue: Linear Mobility. Hysteresis is clockwise as indicated by the arrows. Left: Device with highest mobility at 300°C annealed for four hours in air. Right: Device at 200°C annealed for 12 hours in air. No good working TFT behaviour is observed even after 12 hours of annealing.

Table S1. XPS elemental compositions for samples annealed at 200°C and 300°C for four hours in air. In both cases carbon is a surface contaminant. An estimate of the number of oxygen vacancies from the oxygen ratio is obtained. 7% of oxygen is found to be missing at 300°C. This is an underestimation since surface OH, which are not an intrinsic part of the oxide, have been taken into account in the calculation. Although this value may be different to the true absolute value, the relative values of samples which have the same amount of surface OH can be compared between them. This can be obtained by comparing the value of the bulk OH peak with that of the surface in each sample. The samples annealed at 300°C, 350°C and 400°C which are shown below, had similar surface OH species and could thus be compared. The oxygen vacancy concentration is does not change much with temperature in agreement with other reports. [26] At 200°C the oxygen ratio is larger than one because the oxide is still in its precursor form and the formula O/(1.5In+Zn), which assumes a fully formed oxide, is no longer valid. The oxide ratio is smaller from the expected 1.5. This might indicate that the metal ratio in the film differs from the composition of the solution or that Zn is preferentially detected by XPS.

Name	*Peak*	*At %*
**200°C**		
C1s	284.89	11.92
In3d5	444.62	21.57
O1s	532.05	55.19
Zn2p3	1022.57	11.33
**300°C**		
C1s	284.84	7.33

In3d5	444.58	27.23
O1s	530.17	51.22
Zn2p3	1021.92	14.22

Scan	*Oxygen ratio r_{oxy}*<br>*O/(1.5In+Zn)*<br>*Expected r_{oxy}=1*	*Oxide ratio r_{oxi}*<br>*In_2O_3/ZnO=In/2Zn*<br>*Expected r_{oxi}=1.5*
**Surface 200°C**	1.26	0.95
**Surface 300°C**	0.93	0.96
**Surface 350°C 4h air**	0.91	1.17
**Surface 400°C 4h air**	0.94	1.16

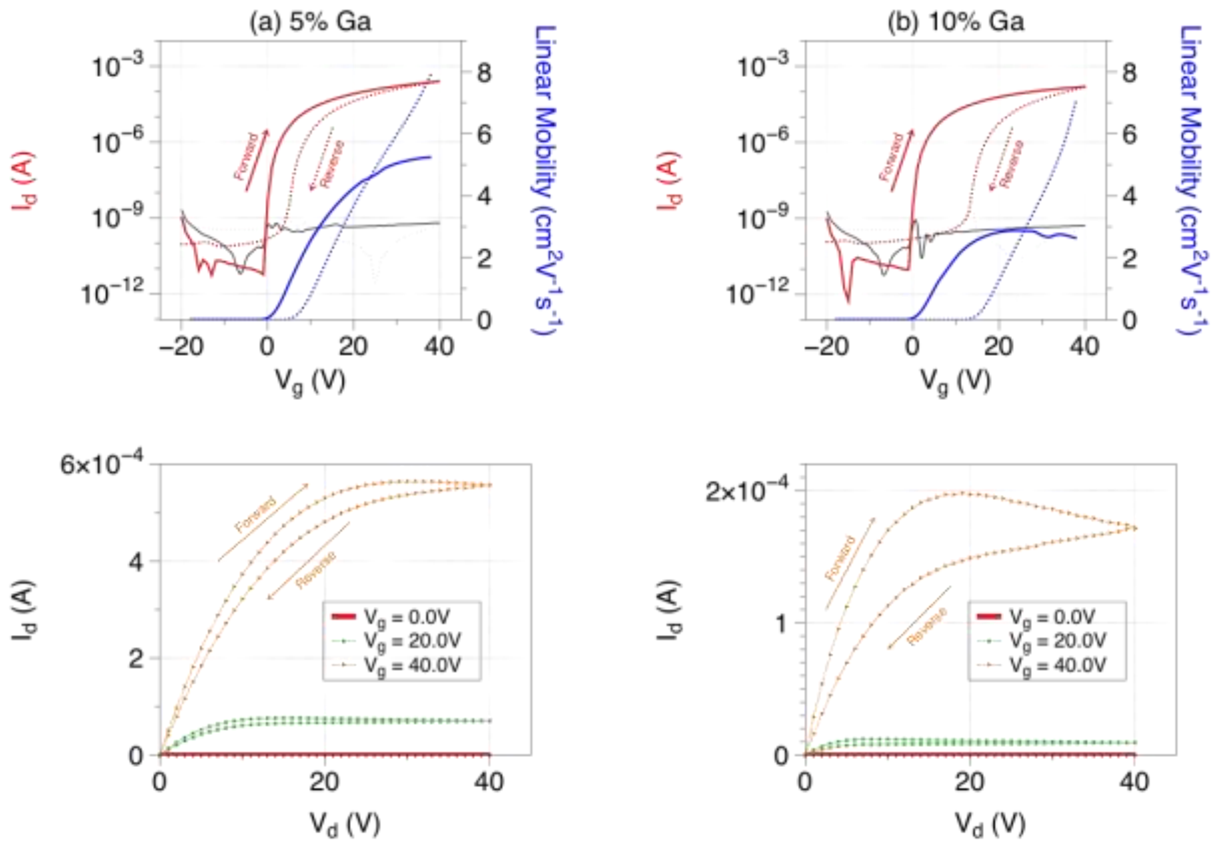

Figure S5. Red: Drain Current. Black: Gate leakage current. Blue: Linear Mobility. Solid lines represent forward scan. Dotted lines represent reverse scan. Transfer (top) and output (bottom) curves for IGZO thin-film transistors: (a) 5% Ga (b) 10% Ga (In_2O_3:ZnO=6:4, W/L=3000μm/300μm, V_d=5V). Films were annealed at 300°C for four hours in air. The

mobility is decreased with increasing Ga doping in agreement with reports that Ga suppresses conductivity through the suppression of donor-like oxygen vacancy formation.[9,11,28-29]

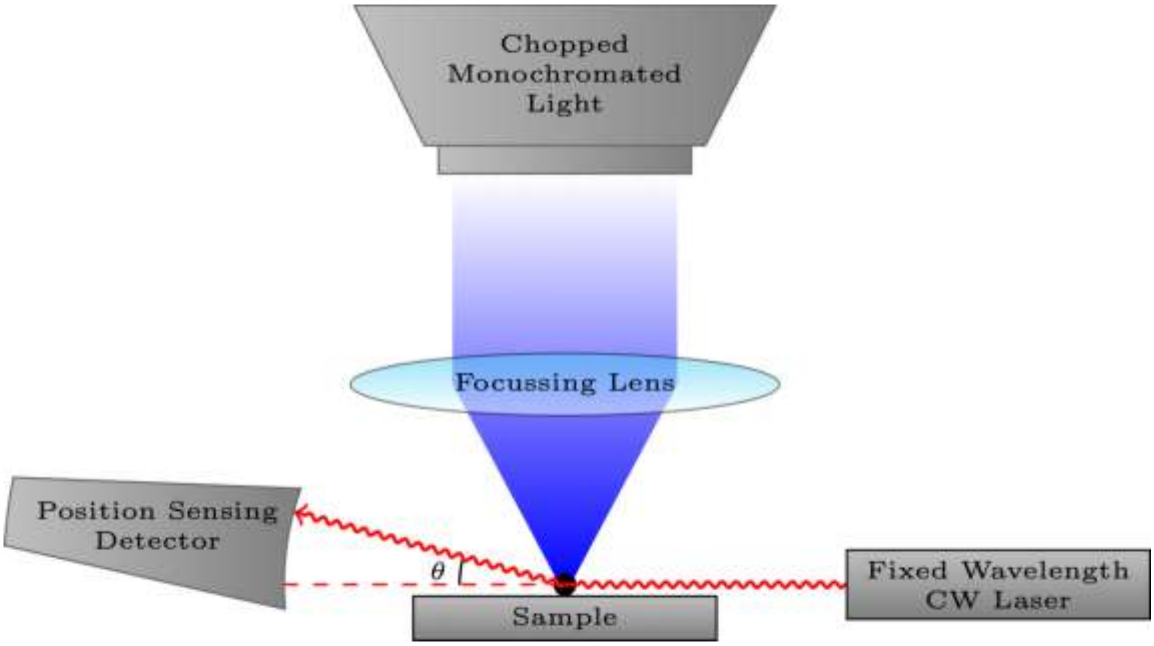

Figure S6. Schematic of a PDS setup. The sample is immersed in a liquid whose refractive index is very sensitive to heat dissipation. Monochromated light is shown onto the sample, which absorbs and hence dissipates some heat. This changes the refractive index of the surrounding liquid. A fixed wavelength laser, directed in parallel to the sample's surface, gets deflected by the refractive index change and this deflection is monitored by a detector. The deflection angle θ is proportional to the absorption in the sample.[32-34]

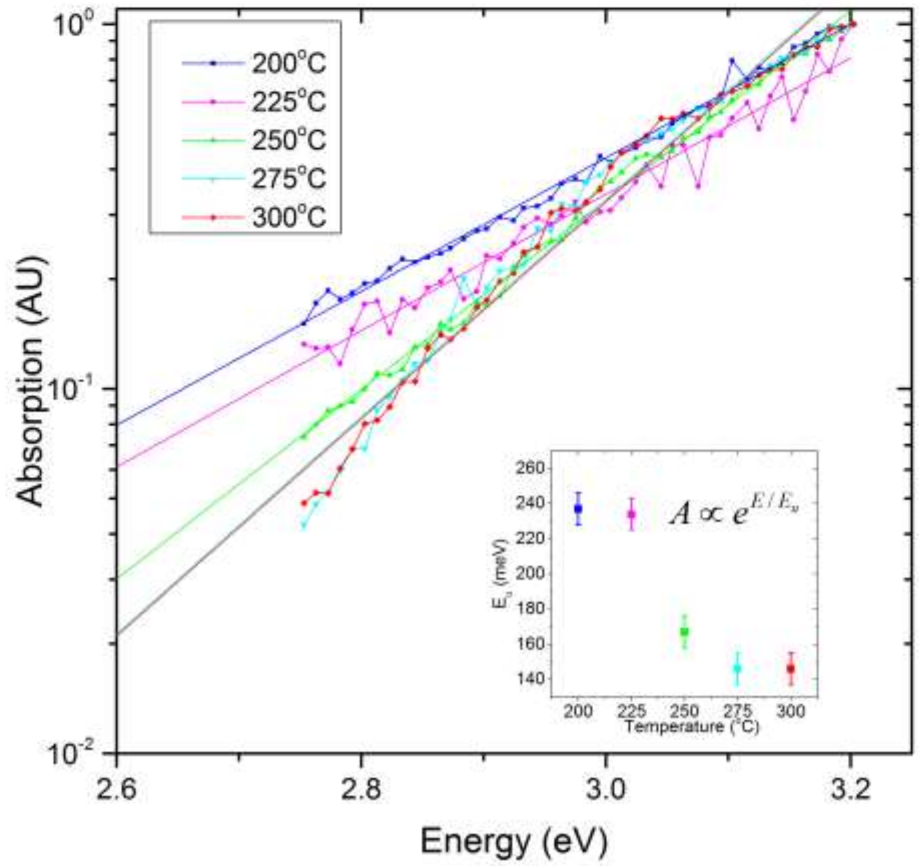

Figure S7. PDS data for samples annealed in air at different temperatures. The data are normalised with respect to the maximum absorption close to the band gap energy ~3.2eV to eliminate any effects arising from different thicknesses between samples. The absorption is within 450meV of the band gap energy and shows the valence band tail. An Urbach energy E_u is calculated using the slope of this tail (E_u=log(e)/slope). This is a measure of the disorder near the VB and is in the range of 140-240meV as shown in the inset.

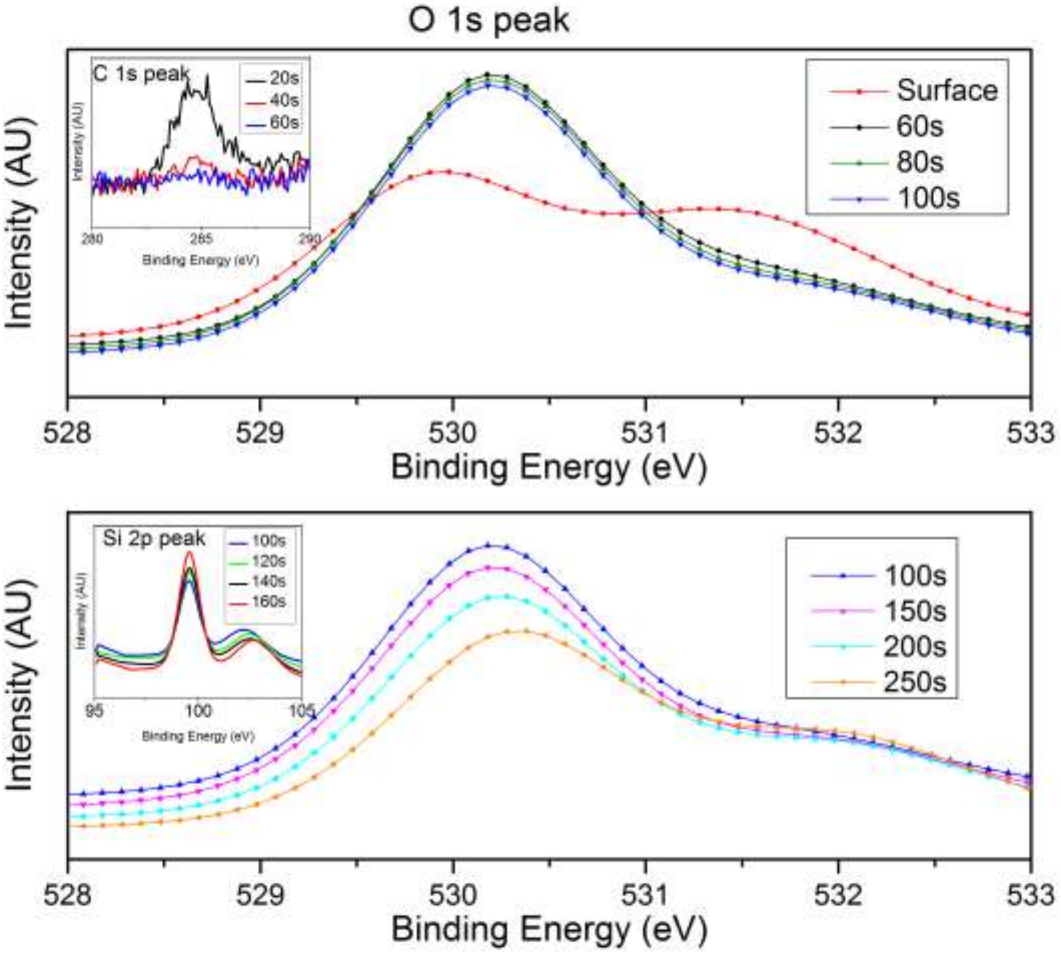

Figure S8. XPS data for oxygen 1s peak after different etching times at an energy of 500eV. The depth profile data are for a film annealed at 300°C for four hours in air. After 60s of etching the OH shoulder at ~531.7eV decreases significantly indicating that the OH surface contamination has been removed. This can also be seen from a scan of carbon which is completely removed after 60s of etching (top inset). At 100s we are still in the bulk of the film. Further etching results in contributions from the silicon substrate as we see the shoulder increasing in intensity and binding energy. We also see this from the silicon 2p peak (bottom inset) which increases. We therefore choose an optimum etching time of 80s which minimizes contributions from the surface contamination or the silicon oxide from the substrate. This analysis is performed on all samples individually to determine the 'bulk' region for each of them.

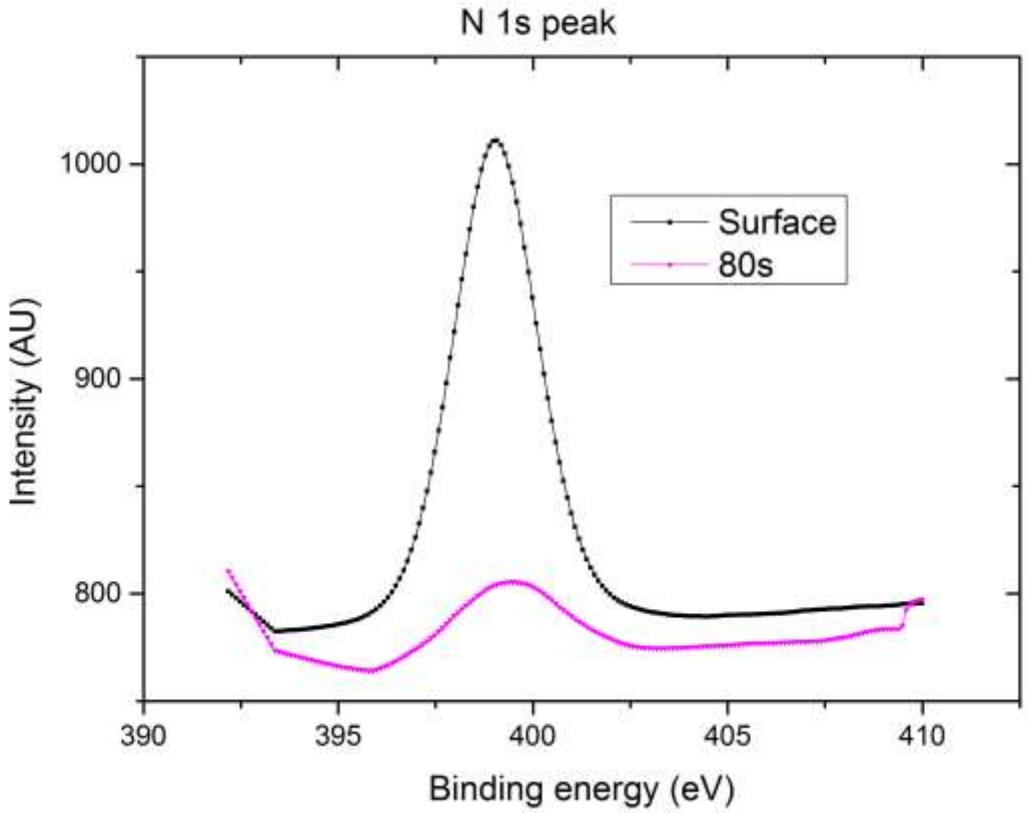

Figure S9. Nitrogen XPS 1s peak for a sample annealed in air at 300°C for four hours. The black curve is a surface scan where nitrogen is detected at 2.26%. The pink curve is taken at the bulk of the oxide and nitrogen is only detected at 0.52% and implies that there are negligible nitrogen residuals in our films. For all other samples the nitrogen peak was at the limits of the XPS and could not be detected even at the surface.

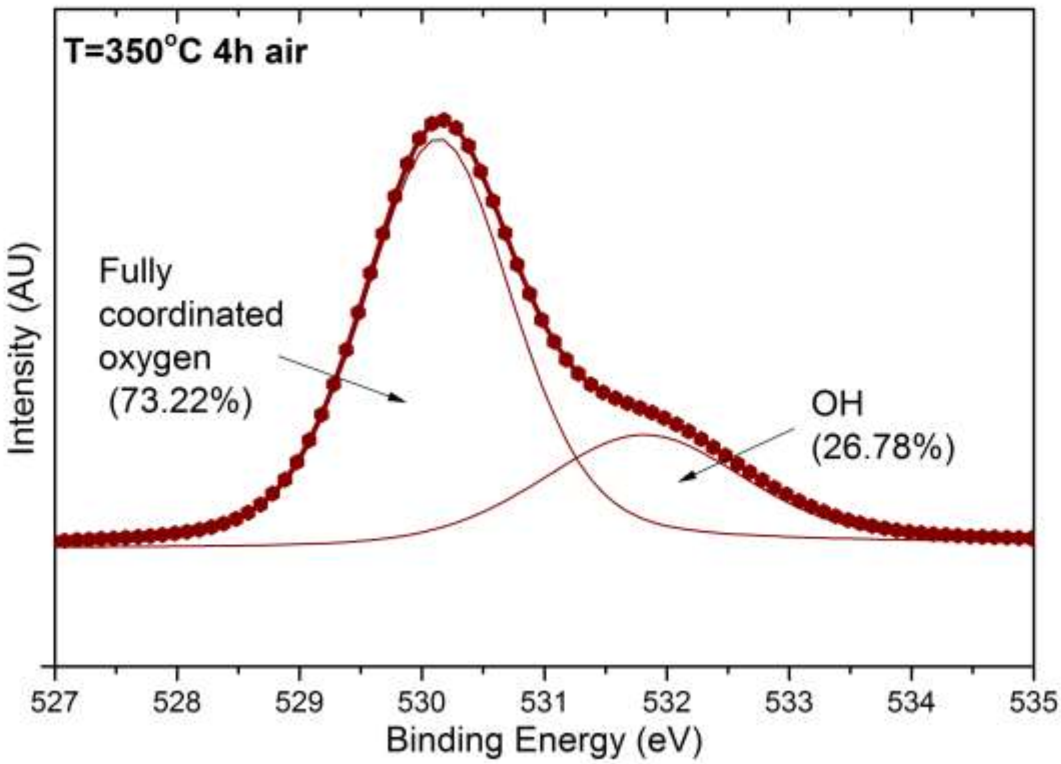

Figure S10. XPS O1s peak for a sample annealed at 350°C in air for four hours. (In_2O_3:ZnO=6:4): We can distinguish two oxygen binding environments: 1) OH species at ~532eV and 2) fully coordinated oxygen at ~530eV.

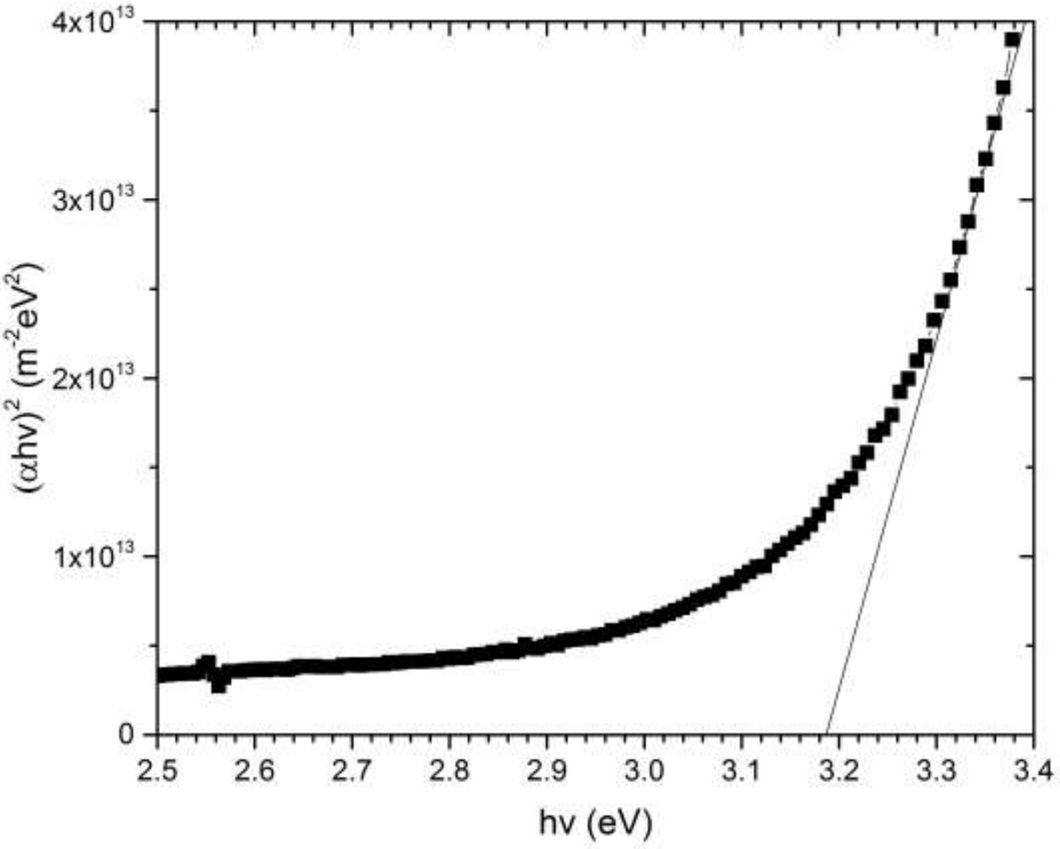

Figure S11. Tauc plot showing an approximate value for the band gap of IZO (In_2O_3:ZnO=6:4). The film was annealed in air for four hours at 300°C. The absorption coefficient α was obtained by dividing the absorbance measured by the film thickness. In order to build a thick layer and enhance the absorption we spin-coated five layers on 15nm quartz spectrosil. Each layer was annealed at 180°C for 10min before spin-coating the next layer. The thickness of one layer is estimated by scanning electron microscopy to be ~10nm and the multiple layers are ~50nm thick. The energy of the incident light is given by hv where h is the Planck constant and v the frequency of the incident photons.

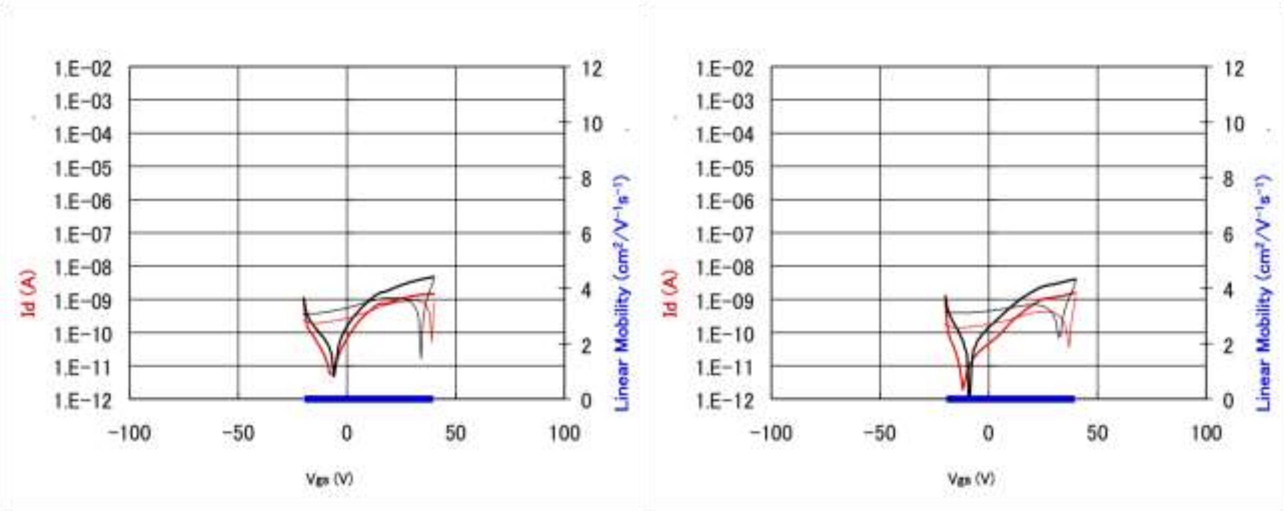

Figure S12. Red: Drain Current. Black: Gate leakage current. Blue: Linear Mobility. Transfer curves for samples annealed at 275°C for four hours in air and subsequently in vacuum at 275°C for one hour (left) and 300°C for 15 minutes (right). No working device is obtained in either case. Annealing for a long time at 275°C can deteriorate the transistor since hydrogen has enough time to diffuse from the interface to the surface. At 300°C even a short annealing time does not produce a good working device implying that hydrogen diffusion occurs quickly at this temperature.

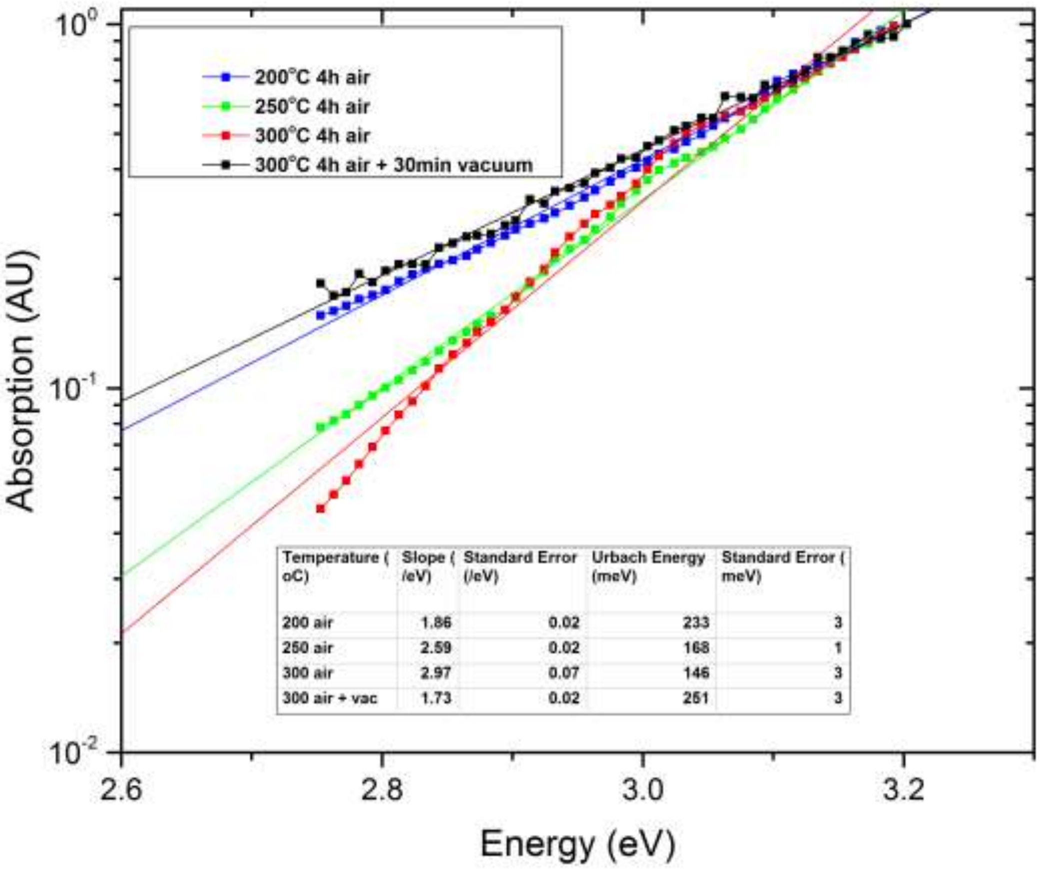

Figure S13. Comparison between PDS data for only air annealed samples at different temperatures and samples with additional vacuum annealing. . The data are normalised with respect to the maximum absorption close to the band gap energy ~3.2eV to eliminate any effects arising from different thicknesses between samples. The absorption is within 450meV of the band gap energy and shows the valence band tail. An Urbach energy E_u is calculated using the slope of this tail (E_u=log(e)/slope). This is a measure of the disorder near the VB and is shown in the inset. The disorder of the vacuum annealed samples is similar to that of the only air annealed sample at 200°C.

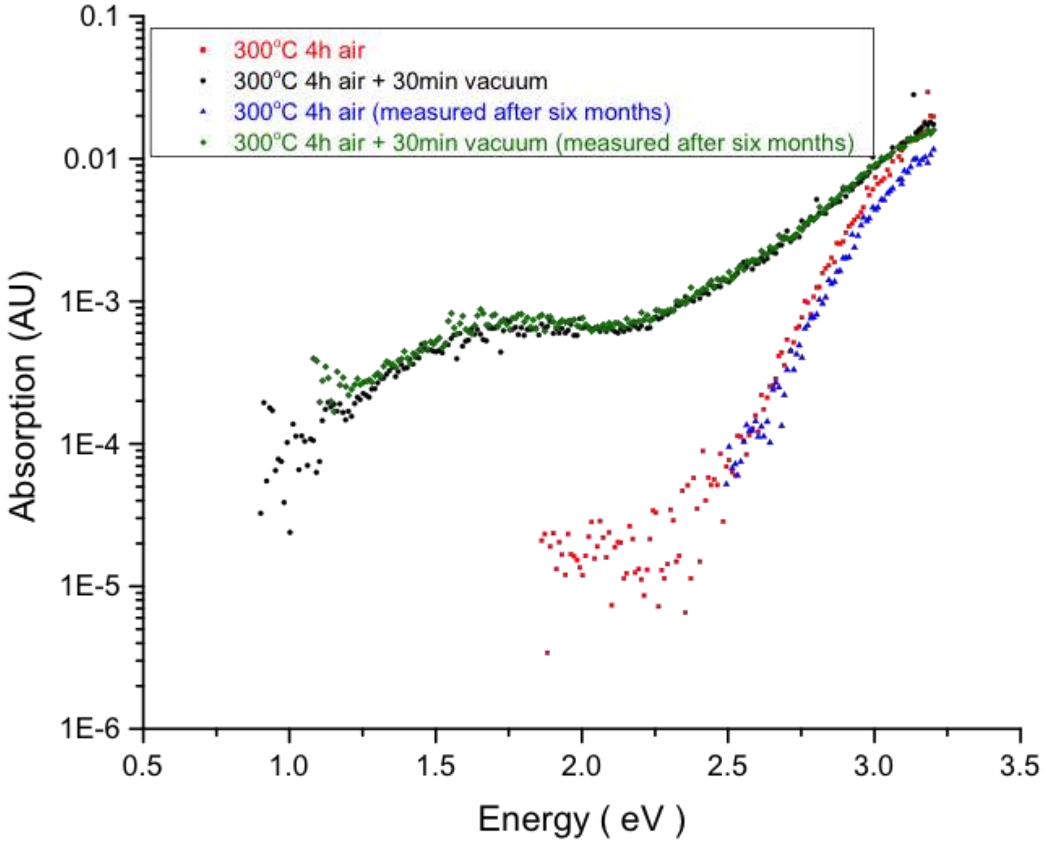

Figure S14. PDS repeated on the same samples after six months showing the stability of the films in air and that transitions between states in the band gap originate from stable bulk defect states.

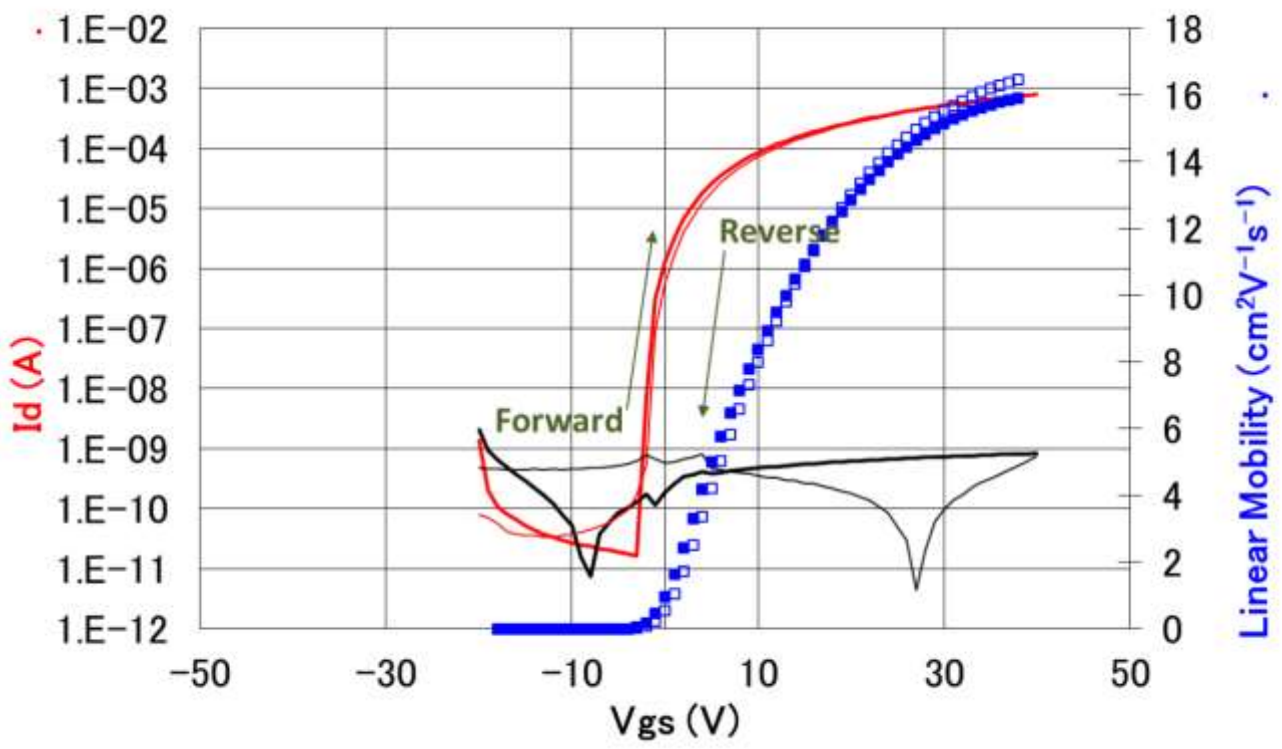

Figure S15. Red: Drain Current. Black: Gate leakage current. Blue: Linear Mobility. Hysteresis is clockwise as indicated by the arrows. The device was annealed in air for four hours at 350°C. The onset voltage starts to shift to negative values V_{on}=-2V since at this high temperature most of the interface and deep traps are filled and the electrons in the conduction band from shallow donors increase the free carrier concentration rather than compensate defect states.

Table S2. Oxygen and oxide ratios for samples with only air anneal and samples with 30 minutes additional vacuum annealing at 275°C and 300°C. Oxygen vacancies seem to be suppressed after vacuum annealing at 275°C and 300°C, by 4% and 3% respectively. These are likely to be donor like vacancies since we know from UPS data that the VB tail for the samples with vacuum annealing increases rather than decreases. In Table S1 the 300°C air annealed samples had 7% missing oxygen whereas here 16% of the oxygen is missing. This difference is due to the different surface OH content since the two samples were fabricated at different periods. The 16% is closer to the true absolute value since this sample contained negligible surface OH. A relative comparison is possible if the samples compared have similar amount of surface OH species. This can be obtained by comparing the value of the bulk OH peak with that of the surface in each sample. The vacuum annealed samples and the only air annealed one at 300°C had almost no surface OH whereas the only air annealed sample at 275°C had a higher surface OH and the oxygen vacancy concentration is underestimated). The oxide ratios show again that the composition in the film is not exactly the same as the composition of the solution (In_2O_3:ZnO=6:4). It should be noted that these analyses can only be done on the surface of the films before any etching has taken place since etching modifies the composition of In and Zn and the calculation becomes unreliable.

Scan	*Oxygen ratio r_{oxy}*<br>*O/(1.5In+Zn)*<br>*Expected r_{oxy}=1*	*Oxide ratio r_{oxi}*<br>*In_2O_3/ZnO=In/2Zn*<br>*Expected r_{oxi}=1.5*
**Surface 275°C 4h air**	0.90	1.11
**Surface 275°C 4h air +30 min vacuum**	0.94	1.17
**Surface 300°C 4h air**	0.84	1.32
**Surface 300°C 4h air + 30 min vacuum**	0.87	1.36
